# Supplementary material for: Adolescent mental health research in Tanzania: a study protocol for a priority setting exercise and the development of an interinstitutional capacity strengthening programme
Source: BMJ Open. 2022 Feb 2;12(2):e054163. doi: 10.1136/bmjopen-2021-054163 (PMC8811585; doi:10.1136/bmjopen-2021-054163)
Supplement: Supplementary data [file bmjopen-2021-054163supp001.pdf]

Scoping questionnaire to inform priority setting for adolescent mental health research in Tanzania:

Researcher Questionnaire v1.3

Study Information:

The National Institute of Medical Research (NIMR) in collaboration with researchers at the Liverpool School of Tropical Medicine, Kilimanjaro Christian Medical University College, and Muhimbili University of Health and Allied Sciences is conducting a research priority setting exercise for adolescent mental health in Tanzania. This involves consulting researchers, implementors and adolescent advocacy groups involved in adolescent mental health.

Why are we contacting you? We are contacting you because we understand that you work in adolescent health and could make an important contribution to this research priority setting exercise.

What do we need from you? A few minutes of your time to complete this short questionnaire about your work and views about adolescent mental health research in Tanzania.

Confidentiality: Your contribution will be treated with the strictest confidence. Your answers will be anonymised before analysis by the research team. Your name or contact information will not be linked to your responses. However at the end of the questionnaire you can leave contact details if you wish to participate in future activities. This contact information will be kept separate from your responses.

Voluntary participation: We would really value your contribution; it will improve the quality of our work. However, your participation is absolutely voluntary and you can withdraw at any time.

How long will the survey take? On average, 15 minutes.

Please feel free to contact us if you would like further information about the project.

Project co-Principal Investigators:

· Dr Ndekya Oriyo, Principal Research Scientist, The National Institute of Medical Research NIMR, Tanzania: ndekya.oriyo@nimr.or.tz

· Dr Angela Obasi, Dept International Public Health, The Liverpool School of Tropical Medicine, UK: angela.obasi@lstmed.ac.uk

---

CONSENT

I have read and understood the information about this survey and understand its purpose.

I understand that no identifiable information about me will be retained from this survey.

I understand that my participation is entirely voluntary and that I can stop or withdraw my participation at any time.

I agree to participate in the survey.

Yes ☐ No ☐

Obasi A, et al. BMJ Open 2022; 12:e054163. doi: 10.1136/bmjopen-2021-054163

ONLINE QUESTIONS for Researcher Scoping Questionnaire

The questionnaire is in two parts. The first few questions are about the work that you are currently doing. The second set of questions ask about what you think are important gaps in adolescent mental health research and service provision.

PART 1: ABOUT THE WORK THAT YOU DO

1. **What is your gender?**
2. **Which age category do you belong to?**
3. **Which institution is your main employer?**

____

4. **Through which institution do you mainly conduct your research?**

____

The next question is about the main focus of your research activities in relation to adolescents

5. **What is your *primary or main* adolescent health research activity?** (Please choose one)**.**
   i. HIV prevention
   ii. HIV treatment and care
   iii. Sexual and reproductive health
   iv. Teenage pregnancy prevention
   v. Teenage pregnancy care
   vi. Adolescent mental health
   vii. Other, please explain

____

The next question is about your adolescent mental health research activities.

6. **MAIN STEM: Are you currently (or have you within the last 5 years been) involved in conducting research addressing adolescent mental health in Tanzania?**
   - Yes
   - No

   If YES (main stem)

7. **What is the primary area of adolescent mental health you are/were researching?**

____

Obasi A, *et al. BMJ Open* 2022; 12:e054163. doi: 10.1136/bmjopen-2021-054163

a. What is/was the objective of this research

____

8. **Which of the following best describes the areas that your <u>adolescent mental health</u> research overall is addressing? Activities are grouped by prevention or treatment.** (Please click all that apply).

   i. Prevention of alcohol use
   ii. Prevention of non-alcoholic substance use
   iii. Prevention of common mental health disorders e.g. depression, anxiety, PTSD
   iv. Prevention of severe mental illness e.g schizophrenia or mania
   v. Prevention of developmental disorders (ADHD, FASD, etc)

   vi. Treatment for alcohol misuse
   vii. Treatment for non-alcoholic substance use
   viii. Treatment of common mental health disorders e.g. depression, anxiety, PTSD
   ix. Treatment of severe mental illness e.g. schizophrenia or mania
   x. Treatment of developmental disorders (ADHD, FASD, etc)

   xi. Other, please explain

9. **What is/was your role in this research**
   a. PI or Co-PI of one or more research projects in this area
   b. Co- investigator of one or more research projects in this area
   c. Research treatment doctor
   d. Research assistant
   e. Other

____

10. **What <u>setting</u> is the primary focus of your studies ? <u>(Please select ONE</u>)**
   b. Adolescents attending mental health services
   c. Adolescents in non-mental health clinical settings
   d. Adolescents in primary school
   e. Adolescents in secondary school
   f. Adolescents attending college
   g. Out of school/community-based
   h. Adolescents in the workplace
   i. Other? Please specify.

11. **In terms of gender, what adolescent subgroup is the primary focus of your research?**
   a. Female adolescents/young people
   b. Male adolescents/young people

Obasi A, *et al. BMJ Open* 2022; 12:e054163. doi: 10.1136/bmjopen-2021-054163

c. Adolescents of any gender

12. What adolescent <u>subgroup</u> is the primary focus of your study? (Please select ONE).

a. HIV positive adolescents
b. Adolescents with disabilities
c. Adolescents who are refugees or seeking asylum
d. Adolescents in domestic service
e. Adolescents who are orphaned
f. Street connected youth
g. Any adolescent
h. Other? [please specify]

____

13. Are there additional adolescents, beyond the primary subgroup chosen above, that your research include? (Please select all that apply)

a. Not applicable
b. Adolescents attending mental health services
c. Adolescents in non mental health clinical settings
d. Adolescents in primary school
e. Adolescents in secondary school
f. Adolescents attending college
g. Out of school/community-based
h. Adolescents in the workplace
i. HIV positive adolescents
j. Adolescents who are orphaned
k. Adolescents with disabilities
l. Adolescents who are refugees or seeking asylum
m. Adolescents in domestic service
n. Street connected youth
o. Other? [please specify]

14. Where is your adolescent mental health research work located? (Click all that apply)

p. [insert all the regions]

____

15. Which of the following best describes your research activity? (click all that apply)

a. Understanding prevalence of mental ill-health among adolescents
b. Exploring risk factors for mental ill-health among adolescents
c. Development or evaluation of prevention interventions
d. Development or evaluation non-pharmacological treatment interventions
e. Development or evaluation pharmacological treatment interventions
f. Adolescent mental health programme evaluation or monitoring
g. Validating measures of adolescent mental ill health
h. Other [please specify]

____

16. Are there any key references, reports or summaries relating to your activities that you could share. If so please could you cite or paste in a weblink here.

Obasi A, *et al. BMJ Open* 2022; 12:e054163. doi: 10.1136/bmjopen-2021-054163

---

13. In addition to your research work are you also involved in the provision of services or care that relate to AMH?
   a. **YES (continue to 14)**
   b. **No – go to the end section**

14. What is your *primary or main* adolescent health activity? (Please choose one)**.**
   i. HIV prevention
   ii. HIV treatment and care
   iii. Sexual and reproductive health
   iv. Teenage pregnancy prevention
   v. Teenage pregnancy care
   vi. Adolescent mental health
   vii. Other, please explain

---

15. Which of the following best describes the type of adolescent mental health activities that you provide? (Click all that apply)
   i. Counselling by trained mental health professionals
   ii. Peer to peer support
   iii. Biomedical treatment
   iv. Behavioural treatment
   v. Education/promotion/prevention
   vi. Other, please explain

Main Stem if Q4 = NO

16. Have research questions relating to adolescent mental health arisen as part of your research activities
- Yes
- No

If yes, please specify

Obasi A, *et al. BMJ Open* 2022; 12:e054163. doi: 10.1136/bmjopen-2021-054163

All stems converge here

PART 2

The next few questions are about what you think are important gaps in adolescent mental health research and service provision

17. Which 3 *areas/issues* in adolescent mental health do you think most urgently need further research?

____

____

____

a. Of these, which is most important for Tanzania as a whole?

____

b. Would you be interested in developing or building collaborations to address any of these research priorities?
- Yes
- No

c. If yes, which area?

____

18. Do you feel that there are gaps in skills/capacity to conduct adolescent mental health research
- **Yes**
- **No**

If yes, which gaps do you feel are the most important? _______________

19. Do you feel that there are gaps in skills/capacity for provision of adolescent mental health care/services?
- **Yes**
- **No**

If yes, which gaps do you feel are the most important? _______________

20. What, if any, networks/groups are you involved in that are conducting or related to Adolescent mental health research in Tanzania?

Obasi A, *et al. BMJ Open* 2022; 12:e054163. doi: 10.1136/bmjopen-2021-054163

21. Would you be interested in participating with us in further adolescent mental health research priority setting activities?

- **Yes**
- **No**

If yes, please provide us with the best email and/or telephone contact for us to reach you

22. Are there any other people working in Adolescent Mental Health that you feel we should approach to ask these questions?

23. Please feel free to use the box below to make any further comments, or share your reflections relating to adolescent mental health research in Tanzania.

END OF QUESTIONNAIRE:

Thank you so much for your participation in this survey

We greatly appreciate your time

For any further queries please contact

ndekya,oriyo@nimr.or.tz or angela.obasi@lstmed.ac.uk
