# Supplementary material for: Adolescent mental health research in Tanzania: a study protocol for a priority setting exercise and the development of an interinstitutional capacity strengthening programme
Source: BMJ Open. 2022 Feb 2;12(2):e054163. doi: 10.1136/bmjopen-2021-054163 (PMC8811585; doi:10.1136/bmjopen-2021-054163)
Supplement: Author's manuscript [file bmjopen-2021-054163.draft_revisions.pdf]

BMJ Open is committed to open peer review. As part of this commitment we make the peer review history of every article we publish publicly available.

When an article is published we post the peer reviewers' comments and the authors' responses online. We also post the versions of the paper that were used during peer review. These are the versions that the peer review comments apply to.

The versions of the paper that follow are the versions that were submitted during the peer review process. They are not the versions of record or the final published versions. They should not be cited or distributed as the published version of this manuscript.

BMJ Open is an open access journal and the full, final, typeset and author-corrected version of record of the manuscript is available on our site with no access controls, subscription charges or pay-per-view fees (http://bmjopen.bmj.com).

If you have any questions on BMJ Open's open peer review process please email info.bmjopen@bmj.com

BMJ Open

Adolescent mental health research in Tanzania - a study protocol for a priority setting exercise and the development of an inter-institutional capacity strengthening programme.

Journal:	*BMJ Open*
Manuscript ID	bmjopen-2021-054163
Article Type:	Protocol
Date Submitted by the Author:	07-Jun-2021
Complete List of Authors:	Obasi, Angela; Liverpool School of Tropical Medicine, International public health<br>Seekles, Maaike; Liverpool School of Tropical Medicine, Department of International Public Health<br>Boshe, Judith; Kilimanjaro Christian Medical Centre, Psychiatry and Mental Health; Kilimanjaro Christian Medical College, Psychiatry and Mental Health<br>Dow, Dorothy; Duke University Medical Center; Kilimanjaro Christian Medical Centre<br>Mmbaga, Blandina; Kilimanjaro Christian Medical Centre<br>Ngakongwa, Fileuka; Muhimbuli National Hospital, Department of Psychiatry and Mental Health<br>Okello, Elialilia; National Institute for Medical Research Mwanza Research Centre, Mwanza Intervention Trials Unit<br>Renju, Jenny; LSHTM; Kilimanjaro Christian Medical University College, Epidemiology and Biostatistics<br>Shayo, Elizabeth; National Institute for Medical Research<br>Simbee, Gema; Mirembe National Psychiatric Hospital<br>Todd, Jim; LSHTM; National Institute for Medical Research Mwanza Research Centre<br>Oriyo, Ndekya; National Institute for Medical Research
Keywords:	MENTAL HEALTH, Child & adolescent psychiatry < PSYCHIATRY, EDUCATION & TRAINING (see Medical Education & Training), Health policy < HEALTH SERVICES ADMINISTRATION & MANAGEMENT, International health services < HEALTH SERVICES ADMINISTRATION & MANAGEMENT

SCHOLARONE™
Manuscripts

Title:

Adolescent mental health research in Tanzania - a study protocol for a priority setting exercise and the development of an inter-institutional capacity strengthening programme.

Corresponding author:

Dr Maaike Seekles
17E Pembroke Place
Liverpool L3 5PH
Maaike.Seekles@lstmed.ac.uk

Authors

Dr Angela Obasi*
Department of International Public Health
Liverpool School of Tropical Medicine
Liverpool, United Kingdom

Dr Maaike Seekles*
Department of International Public Health
Liverpool School of Tropical Medicine
Liverpool, United Kingdom

Dr Judith Boshe
Kilimanjaro Christian Medical University College
Moshi, Tanzania

Dr Dorothy Dow
Department of Pediatrics, Infectious Diseases,
Duke University
Durham, North Carolina, USA

Professor Blandina Mmbaga
Kilimanjaro Clinical Research Institute
Moshi, Tanzania

Dr Fileuka Ngakongwa
Department of Psychiatry and Mental Health
Muhimbuli National Hospital
Dar es Salaam, Tanzania

Dr. Elialilia Okello
Mwanza Intervention Trials Unit
NIMR Mwanza Research centre, Mwanza, Tanzania

For peer review only

Dr Jenny Renju
Department of Epidemiology and Biostatistics,
Kilimanjaro Christian Medical University College
Moshi, Tanzania

Dr Elizabeth H. Shayo
Department of Policy Analysis and Advocacy
National Institute for Medical Research
Dar es Salaam, Tanzania

Dr Gema Simbee
Mirembe National Psychiatric Hospital
Dodoma, Tanzania

Professor Jim Todd
Department of Population Health
London School of Health and Tropical Medicine
London, United Kingdom

Dr Ndekya Oriyo†
National Institute for Medical Research
Dar es Salaam, Tanzania

***** Joint first authors

† Senior Author

Word count: 3424 words (incl declarations, excl abstract, figures, tables and references)

For peer review only

For peer review only - http://bmjopen.bmj.com/site/about/guidelines.xhtml

Abstract

Introduction

Poor adolescent mental health is a barrier to achieving several sustainable development goals in Tanzania, where adolescent mental health infrastructure is weak. This is compounded by a lack of community and policy maker awareness or understanding of its burden, causes and solutions. Research addressing these knowledge gaps is urgently needed. However, capacity for adolescent mental health research in Tanzania remains limited. The existence of a National Institute for Medical Research (NIMR), with a nationwide mandate for research conduct and oversight, presents an opportunity to catalyse activity in this neglected area. Rigorous research priority setting, which includes key stakeholders, can promote efficient use of limited resources and improve both quality and uptake of research by ensuring that it meets the needs of target populations and policy makers. We present a protocol for such a research priority setting study and how it informs the design of an inter-institutional adolescent mental health research capacity strengthening strategy in Tanzania.

Methods and analysis

From May 2021, this six-month mixed-methods study will adapt and merge the James Lind Alliance approach and validated capacity strengthening methodologies to identify priorities for research and research capacity strengthening in adolescent mental health in Tanzania. Specifically, it will use online questionnaires, face-to-face interviews, focus groups, scoping reviews and a consensus meeting to consult expert and adolescent stakeholders. Key evidence-informed priorities will be collaboratively ranked and documented and an integrated strategy to address capacity gaps will be designed to align with the Tanzania National Institute for Medical Research strategy and nationwide infrastructure.

Ethics and Dissemination

For peer review only

For peer review only - http://bmjopen.bmj.com/site/about/guidelines.xhtml

The study was approved by both NIMR and Liverpool School of Tropical Medicine Research Ethics Committees. Results will be disseminated through a national workshop involving all stakeholders, through ongoing research and capacity strengthening collaborations and through published commentaries, reviews, policy briefs, webinars and social media.

Key words: Mental health, adolescent health, capacity strengthening, research priority setting, Tanzania, James Lind Alliance

For peer review only

For peer review only - http://bmjopen.bmj.com/site/about/guidelines.xhtml

Strengths and limitations of this study

1. Systematic inclusion of all stakeholders, including policy makers
2. Nationwide approach and dissemination strategy using NIMR infrastructure
3. Transparent and integrated approach to research priority setting and capacity strengthening
4. Inclusion of adolescent stakeholders aged 18+ limits applicability to younger adolescents at this stage but will be the focus of future activity

For peer review only

For peer review only - http://bmjopen.bmj.com/site/about/guidelines.xhtml

INTRODUCTION

Mental health disorders account for a high burden of disease among young people worldwide. They are responsible for three of the top six causes of disability adjusted life years lost among 10-24 year olds [1] and affect up to 20% of all adolescents.[2] Suicide is the third leading cause of death in this age group. Adolescents who experience depressive disorders and other mental health problems are more likely to engage in risky behaviours, such as unprotected sexual activity and substance misuse. They are also more likely to have low educational attainment, to be unemployed and to live in poverty.[3] Much of adult mental ill-health begins in adolescence, with an estimated 75% of all lifetime mental disorders occurring by the age of 25.[4] The burden of mental ill-health among adolescents and its potential impact on adult outcomes makes adolescence a critical time for mental health promotion, early identification of mental health disorders, and rapid access to effective adolescent mental health (AMH) care.[2, 5, 6] This is especially important in Africa, which will be home to around 35 per cent of the world's adolescents by 2050.[7]

In Tanzania, where around half of the population is under 20 years of age, there are several important barriers to effective prevention and care for AMH disorders.[8] First, research on the prevalence, causes and solutions for several AMH issues is lacking.[9,10] The few available studies, mainly on depression and alcohol use disorders, indicate that AMH issues are common in Tanzania.[8,11] Second, AMH disorders are often not recognised as illnesses and stigmatisation of mental ill-health presents a further barrier to care.[12,13] Third, AMH is a low policy priority.[14] In 2017, there were 0.06 psychiatrists and 0.01 psychologists per 100,000 population; this is well below average for the Africa Region.[15] Currently, Tanzania has only one psychiatrist specialised in child and AMH disorders (Dr. Gema Simbee, 2021). Further, primary and community-based healthcare providers, such as community health workers, general nurses, and community health officers, often have received very limited training, if any, in the delivery of mental healthcare to adolescents.[16, 17]

For peer review only - http://bmjopen.bmj.com/site/about/guidelines.xhtml

Health research is an essential tool for addressing health and development challenges and for informing health policy. Careful prioritisation of research to maximise its likely impact and the efficient use of resources is especially important where resources are scarce.[18] Of equal importance is the capacity to conduct high quality research to address identified priorities. Limited research capacity in low and middle income countries (LMIC) is a recognised barrier to improved health and health systems.[19–21] Research capacity in Tanzania faces both significant challenges and opportunities.[22] In particular, the existence of a national, publicly funded Institute for Medical Research provides the opportunity for systematic, nationwide scale up of both research and capacity strengthening. With eight research centres across Tanzania, The National Institute for Medical Research (NIMR) is the largest research Institute in Tanzania. It is mandated by government to conduct, oversee, and drive health research nationally, develop national health research priorities and oversee research implementation.

Against this background, the present study will first conduct rigorous research priority setting (RPS) to identify current AMH research gaps and priorities in Tanzania. In line with best practice, perspectives of policy makers, implementers and adolescent advocates will be thoroughly canvassed to ensure priorities meet the needs of all stakeholders. This will include mapping of AMH service implementation within the Government and civil society sector, including professional associations and internationally funded programmes. Second, we will concurrently map and identify gaps in existing AMH research capacity. This will inform the development of a strategy to ensure sufficient research capacity to address identified research gaps and priorities, including optimal use of the opportunity provided by NIMR and other research institutions and Civil Society Organisations (CSOs) within the country.

Study aims and objectives

The study aims are two-fold: first to identify gaps and priorities in AMH research in Tanzania which will inform the co-development of an evidence-based, stakeholder-driven research agenda; and

For peer review only - http://bmjopen.bmj.com/site/about/guidelines.xhtml

second to identify gaps in capacity needed to deliver this research agenda which will inform the development of a national research capacity strengthening (RCS) programme to address these gaps.

The specific research objectives are as follows:

1. Identify and bring together leading researchers, implementers and agencies, policy and decision-makers, and other AMH stakeholders in Tanzania.
2. Summarise and catalogue available evidence on the AMH burden and on the implementation and evaluation of AMH interventions in Tanzania.
3. Identify research priorities and develop a future research agenda for AMH.
4. Map existing research capacity and identify gaps in capacity needed to deliver the research agenda.
5. Design an RCS programme to address AMH research priorities.

METHODS AND ANALYSIS

Rationale for selection of methodologies

There is considerable variation in approaches, tools and methods used in health RPS.[23] Recent WHO RPS guidance[24] identifies four phases from planning to evaluation (Figure 1) and discusses a range of tools and approaches which can be used in each phase. The guidance recommends use of comprehensive approaches to improve the quality and transparency of the exercise.[24] One widely-recommended approach, which has been used previously in Africa,[25] is a methodology designed by the James Lind Alliance (JLA).[26]

Insert here: Figure 1. The RPS process. Reproduced with permission from World Health Organization,[24] licence: CC BY-NC-SA 3.0 IGO.

Well-described and freely available, it consists of four steps which are aligned to the planning and implementation phases of the WHO cycle as follows (See Figure 2):

For peer review only - http://bmjopen.bmj.com/site/about/guidelines.xhtml

1. Initiation: Establish a group of stakeholders.
2. Gathering uncertainties (consultation): Via a questionnaire, stakeholders are asked to present their list of possible research priorities.
3. Verifying uncertainties (collation): The research team theme the possible priorities. A literature search is done to check whether there is any existing research evidence on the possible priorities.
4. Prioritisation: During a priority setting workshop, involving discussions in break-out groups and the stakeholder group as a whole, a top 10 of research priorities will be agreed upon.

The current research will adapt and build on the JLA methodology and combine it with activities related to RCS as the second component of this study.

Our RCS will use methods recommended by Bates et al. which have been validated in a number of research institutions in Africa [27] and consist of the following steps,[27] the first four of which fall within the scope of this study:

1. Define the goal of the capacity strengthening programme.
2. Describe the required capacity needed to achieve the goal.
3. Determine the existing capacity and identify any gaps compared to the required capacity.
4. Devise and implement an action plan to fill the gaps, setting out activities, cost implications and indicators of progress.
5. Learn through doing; adapt the plan and indicators regularly.

This protocol details the application of the RPS and the modification of the RCS methods and processes that will be implemented to achieve the study objectives.

Detailed Study Design

This mixed methods study will be conducted over six months in four overlapping phases and will include online questionnaires, scoping reviews of the literature, individual interviews, focus group

For peer review only

discussions and workshops. RPS and RCS activities will run concurrently where appropriate (See figure 2). This protocol has been prepared using the Standard Protocol Items: Recommendations for Interventional Trials Guidelines (SPIRIT, see supplementary file 1), where applicable.

Participants

All stakeholders in the field of AMH in Tanzania will be eligible to participate if they are 18 years of age or older. This includes active researchers, implementers, agencies, youth advocacy groups, and any relevant policy makers. For logistical reasons including prevailing COVID-19 restrictions and the lack of resources needed to create safe spaces for younger adolescents currently receiving treatment, adolescents aged below 18 years could not be included. However, this study will inform the design of a future priority setting activity that will focus specifically on younger adolescent services users and their carers.

Patient and public involvement

This preparatory consultation study is constrained by limited resources, the challenges of the ongoing COVID-19 pandemic and the complexities related to access to vulnerable AMH service users. The study largely applies the methods as described in the JLA RPS methodology, but departs from the approach in terms of study population. A JLA RPS exercise typically involves patients, carers and clinicians.[26] Our study is limited to professional stakeholders and youth advocates aged 18 or over. As explained above, engaging younger patients is outside the scope of this study, but will be a main subject of investigation in future follow-on studies when Covid-19 constraints have been relaxed and funds are available to ensure safety and support. This study will, however, gain the input from young people and service users in the community, by including representatives of youth advocacy groups, whose expert views are of pivotal importance.

Insert here: Figure 2. Project flow diagram.

For peer review only - http://bmjopen.bmj.com/site/about/guidelines.xhtml

Processes

Phase 1: Identification and recruitment of stakeholders (February – June 2021)

The initiation phase aims to identify and bring together stakeholders in the field of AMH. As a starting point, the research team, which includes researchers from NIMR, Kilimanjaro Christian Medical University College, Research Institute and Medical Centre and Muhimbili University of Health and Allied Sciences, will list any active researchers, implementers and agencies, and youth advocacy groups that they are aware of in Tanzania. In addition, relevant policy makers working in the field of AMH will be identified. Any further stakeholders who are identified will be offered the opportunity to join the study, making the identification of stakeholders an iterative process throughout.

Phase 2: Initial consultation (Spring 2021)

The second phase aims to 'gather uncertainties'[26] and gain an initial insight into stakeholder views on priorities and capacity gaps in AMH research.

Insert here: Figure 3. Survey and workshop recruitment flow chart.

Questionnaire

The research team will distribute a link to an on-line questionnaire (using Survey Monkey) to all identified stakeholders. It is expected that around 60 participants will be reached. A sample size calculation was not appropriate, since all identified stakeholders will be invited to participate. The questionnaire will include a consent statement, followed by a mix of open-ended and closed questions. The questions will be adapted slightly by type of stakeholder, but will generally address current involvement in the field of AMH service provision or research; perceived gaps in AMH research skills/capacity; and gaps in provision of AMH care. In addition, participants are asked to list three AMH research priorities. To ensure that all active stakeholders are reached through a process of snowballing, respondents will be asked to

For peer review only

For peer review only - http://bmjopen.bmj.com/site/about/guidelines.xhtml

nominate other relevant stakeholders. Respondents will also be invited to signal their willingness to participate in the remainder of the RPS and RCS process. The data collection will take place over a period of one month commencing May 2021. In case of non-response, to maximise participation, identified stakeholders will receive two reminders via e-mail. Because of the high likelihood that stakeholders not supported by research infrastructure will have poor internet connectivity, non-researchers will receive reminders via phone calls and/or a text message. The study will also be publicised through research and implementer networks (see figure 3).

A small number of key informant interviews (n~5-10) and focus group discussions (e.g., with representatives of national professional organisations and youth advocates) will take place, addressing emergent case stories or themes. Focus groups and interviews, conducted in either English or Kiswahili, will conform to prevailing COVID-19 social distancing policies and be conducted remotely where necessary. They will be recorded and transcribed verbatim.

Phase 3: Scoping reviews (Spring/Summer 2021)

The JLA and Bates et al. recommend the use of a literature review in an early stage of RPS and RCS exercises.[26, 27] A scoping review was found to be the most appropriate method for this step in the current study. A scoping review does not aim to produce a critically appraised and synthesised answer to a precise question related to a certain treatment or practice. It rather aims to provide an overview or map of the available evidence.[28] It can report on the types of evidence that address and inform AMH practice in Tanzania and the way research has been conducted, which aligns with the interests of the research team. The scoping reviews will follow the framework of Arksey and O'Malley.[29] The research questions, search terms and inclusion criteria underpinning this review will be informed by research priorities and capacity gaps identified in the questionnaire. In addition to issues emerging from the survey and allied activities, scoping reviews will address: Indicators, measurement tools and definitions of moderate to severe AMH disorders;[30] mHealth and digital innovations in AMH;[31] Marginalized and vulnerable adolescents;[30, 32] AMH literacy and stigma

For peer review only - http://bmjopen.bmj.com/site/about/guidelines.xhtml

across sectors;[8, 16, 30] AMH health system strengthening and multisectoral engagement;[8, 33] Ethics of AMH research and implementation;[34] and Gender and AMH,[34, 35] which were already identified as priorities during the preparatory research conducted in the design phase of this study.

Phase 4: Prioritisation and RCS programme development (Summer 2021)

The final phase aims to synthesise the information collected through the above activities and bring stakeholders together to design a research agenda and develop an RCS programme.

Workshop

After analysis of the questionnaire and initial scoping reviews a workshop will synthesise research priorities and refine the goal for the RCS programme. Participants will be selected from the larger stakeholder group based on their availability and whether they registered an interest to take part. It is estimated that around 25 stakeholders, including national and sub-national representatives of the Ministry of Health and local government, will be able to participate. This falls within the range recommended by the JLA of 12-30 participants per workshop.[26] Should more than 30 stakeholders want to participate, the research team will purposively select participants to ensure an appropriate representation of all stakeholder groups. During the two-day workshop, group discussions will take place, focusing on current research and perceived gaps in research, research capacity and care for AMH.

Per JLA guidance, an adapted Nominal Group Technique will be used as the method for research priority setting. Participants will first form smaller discussion groups of maximum 8 participants, during which each participant gives their view about which priorities are most and least important, followed by shared ranking or voting. The ranked orders for each item from each group are totalled, and the priority with the lowest, i.e., most favoured, total ranking is selected as the top priority. The ranking is then discussed and reviewed in the large group, to arrive at a final list of 10 research priorities.[26] The final

For peer review only - http://bmjopen.bmj.com/site/about/guidelines.xhtml

priorities will inform the development of a research agenda on AMH, with key research questions outlined in collaboration with partners.

The remainder of the workshop will focus on RCS. Informed by findings from individual interviews, FGD and the scoping review, the capacity needs assessment will be finalised. During group discussions, a process of joint problem-solving with stakeholders will identify strengths, prioritise critical capacity gaps, and transform what evidence indicated was optimal into what is feasible and practical. The gaps, reasons for the gaps, discrepancies, and resolutions, and potentially sustainable solutions for filling capacity gaps are mapped onto a matrix.[27] The team, together with participants, will then use this matrix to underpin the design of a theory of change and an RCS programme. The RCS strategy will align activities for strengthened capacity, e.g. mentoring, PhD in specific areas, skills development to potential studies and study partnerships, with identified research priorities. Intra and inter-institutional partnerships and additional resources needed to deliver these activities will be agreed.

Ethics and Dissemination

National and Institutional review board approvals were sought and granted from the National Health Research Ethics Committee of the NIMR Medical Research Coordinating Committee in Tanzania (Reference number: NIMR/HQ/R.8a/Vol. IX/3670) and the LSTM in the United Kingdom (Reference number: 21-002).

Participation in this study is voluntary. All stakeholders will receive information about the study, to allow them to make an informed decision about their participation. Survey participants are asked to confirm approval with a consent statement. At the workshop, participants will be asked to sign an informed consent form. They have the right to withdraw at any time by informing the Principal Investigator. However, participants are advised that once their data is anonymised, it cannot be removed. Due to the nature of the focus group discussions and workshops, confidentiality cannot be guaranteed, but participants will be reminded to not share any sensitive data that can bring harm upon themselves or others.

For peer review only

A COVID-19 Risk Assessment, detailing appropriate precautions to limit the spread of COVID-19, has been completed. This will be updated throughout the study duration, to ensure that research activities comply with latest regulations.

A national dissemination meeting will be organised in Tanzania once the strategy and priority setting is finalised and written up, during which the results of the RPS exercise and RCS assessment will be presented. The developed national AMH research agenda will be launched as a platform for collaborative, inter-institutional research programmes which will embed RCS activities. The findings will be further disseminated through commentaries, reviews, and policy briefs.

Data management and analysis

This study will comply with LSTM and NIMR data protection policies.[36,37] All electronic data and personal information of participants will be stored on password protected computers and secure drives, only accessed by designated members of the research team as appropriate. Group discussions and interviews will be recorded on an encrypted device; recordings will be transcribed verbatim as soon as possible after data collection, after which recordings will be deleted. Data will be stored for a maximum of 10 years, to allow verification of data from external sources if necessary, or longer if used for further research.

Data from online questionnaires will be extracted from Survey Monkey and simple descriptive statistical analysis will be undertaken, using Access or STATA software to provide summaries of the sample and responses. In addition, answers to open questions will be coded and analysed using content analysis to arrive at an initial list of gaps in research and capacities.

Workshop data and data collected during group discussions and interviews will be thematically analysed, using NVivo software, to offer a more in-depth account of arguments for or against research priorities, and explanations and possible solutions for capacity constraints and research gaps.

DISCUSSION

For peer review only - http://bmjopen.bmj.com/site/about/guidelines.xhtml

To summarise, this study will use a mixed-methods approach, to consult experts, policy-makers and advocates and identify priorities and capacity gaps for AMH research in Tanzania. It gains its strengths from a multidisciplinary partnership between, and the involvement from inception of, key stakeholders across a number of research institutions across Tanzania; the use of a systematic, transparent approach to RPS; an evidence-based approach to RCS which is aligned to priority research activities; and a strong commitment to dissemination.

As discussed earlier, this study departs from the JLA method in terms of study population. It also departs from the approach in terms of scope of investigation. The JLA approach usually focuses on uncertainties around treatment for a specific illness.[26] The aim of this study is to identify priorities on a national level, across the spectrum of AMH research. This could include uncertainties around prevalence, prevention, and treatment of varying illnesses, in addition to wider systemic challenges. By engaging key policy makers and the leadership and structure of the NIMR, it is hoped this study will raise the profile of AMH research within national research policy. The inter-institutional RCS partnerships generated through this process are also aligned to other best practice strategies such as the WHO ESSENCE guidance.[38] In particular, it is envisaged that the opportunities provided for AMH researchers, representatives from civil society organisations, development partners, youth advocates and senior government to meet and network during this study, will build the foundations for a strong AMH research structure and legacy.

For peer review only

DECLARATIONS

Consent for publication

Not applicable.

Competing interests

The authors declare that they have no competing interests.

For peer review only - http://bmjopen.bmj.com/site/about/guidelines.xhtml

Author's contributions

AO and NO are co- principal investigators who jointly initiated and led design of the study and share overall responsibility for the research. All authors have since contributed to the design of the study and data collection materials.

AO and MS led the drafting of the paper. All authors contributed to drafting of the manuscript and commented on the final version.

Funding

This study is funded by a networking grant from the Global Challenges Research Fund (UKRI, Ref: GCRFNGR6_1454). The funder has no involvement in the design; collection, management, analysis, and interpretation of data; writing of the report, and the decision to submit the report for publication, nor do they have ultimate authority over any of these activities.

REFERENCES

1 Abbafati C, Machado DB, Cislaghi B, *et al.* Global burden of 369 diseases and injuries in 204 countries and territories, 1990–2019: a systematic analysis for the Global Burden of Disease Study 2019. *Lancet* 2020;**396**:1204–22. doi:10.1016/S0140-6736(20)30925-9

2 World Health Organization. Adolescent Mental Health: Time for Action. 2019. https://www.who.int/pmnch/knowledge/publications/AMH.pdf?ua=1 (accessed 1 Dec 2020).

3 Nyundo A, Manu A, Regan M, *et al.* Factors associated with depressive symptoms and suicidal ideation and behaviours amongst sub-Saharan African adolescents aged 10-19 years: cross-sectional study. *Trop Med Int Heal* 2020;**25**:54–69. doi:10.1111/tmi.13336

4 Kessler RC, Amminger GP, Aguilar-Gaxiola S, *et al.* Age of onset of mental disorders: A review of recent literature. Curr. Opin. Psychiatry. 2007;**20**:359–64.

For peer review only - http://bmjopen.bmj.com/site/about/guidelines.xhtml

doi:10.1097/YCO.0b013e32816ebc8c

5 World Health Organization. mhGAP Mental Health Gap Action Programme. World Health Organization 2013. https://www.who.int/mental_health/evidence/mhGAP/en/ (accessed 10 Dec 2020).

6 World Health Organization. Global Accelerated Action for the Health of Adolescents (AA-HA!) Guidance to Support Country Implementation. 2017. https://apps.who.int/iris/bitstream/handle/10665/255415/9789241512343-eng.pdf?sequence=1 (accessed 10 Dec 2020).

7 UNICEF. Generation 2030 Africa 2.0 . 2017. https://data.unicef.org/resources/generation-2030-africa-2-0/ (accessed 26 Jan 2021).

8 Kutcher S, Perkins K, Gilberds H, *et al.* Creating Evidence-Based Youth Mental Health Policy in Sub-Saharan Africa: A Description of the Integrated Approach to Addressing the Issue of Youth Depression in Malawi and Tanzania. *Front Psychiatry* 2019;**10**:542. doi:10.3389/fpsyt.2019.00542

9 Cortina MA, Sodha A, Fazel M, *et al.* Prevalence of child mental health problems in Sub-Saharan Africa: A systematic review. Arch. Pediatr. Adolesc. Med. 2012;**166**:276–81. doi:10.1001/archpediatrics.2011.592

10 Kieling C, Baker-Henningham H, Belfer M, *et al.* Child and adolescent mental health worldwide: evidence for action. *Lancet* 2011;**378**:1515–25. doi:10.1016/S0140

11 Francis JM, Weiss HA, Mshana G, *et al.* The epidemiology of alcohol use and alcohol use disorders among young people in Northern Tanzania. *PLoS One* 2015;**10**. doi:10.1371/journal.pone.0140041

12 Iseselo MK, Kajula L, Yahya-Malima KI. The psychosocial problems of families caring for relatives with mental illnesses and their coping strategies: A qualitative urban based study in

For peer review only - http://bmjopen.bmj.com/site/about/guidelines.xhtml

Dar es Salaam, Tanzania. *BMC Psychiatry* 2016;**16**:1–12. doi:10.1186/s12888-016-0857-y

13 Benedicto M, Mndeme E, Mwakagile DSM, *et al.* Community Knowledge, Attitudes and Perception towards Mental Illness in Dodoma Municipality, Tanzania. *ARC J Public Heal Community Med* 2016;**1**:10–8. doi:10.20431/2456-0596.0103002

14 Zhou W, Ouyang F, Nergui O-E, *et al.* Child and Adolescent Mental Health Policy in Low- and Middle-Income Countries: Challenges and Lessons for Policy Development and Implementation. *Front Psychiatry* 2020;**11**:150. doi:10.3389/fpsyt.2020.00150

15 World Health Organization. Mental Health Atlas. 2018. https://apps.who.int/iris/bitstream/handle/10665/272735/9789241514019-eng.pdf?ua=1 (accessed 26 Jan 2021).

16 Kutcher S, Wei Y, Gilberds H, *et al.* Addressing Adolescent Depression in Tanzania: Positive Primary Care Workforce Outcomes Using a Training Cascade Model. *Depress Res Treat* 2017;**2017**:1–9. doi:10.1155/2017/9109086

17 World Health Organization. Mental Health Atlas 2017 - Republic of Tanzania. 2017. https://www.who.int/mental_health/evidence/atlas/profiles-2017/TZ.pdf?ua=1 (accessed 25 Nov 2020).

18 McGregor S, Henderson KJ, Kaldor JM. How are health research priorities set in low and middle income countries? A systematic review of published reports. *PLoS One* 2014;**9**:1–9. doi:10.1371/journal.pone.0108787

19 Council on Health Research for Development. Health Research: Essential Link to Equity in Development . 1990. http://www.cohred.org/publications/open-archive/1990-commission-report/ (accessed 11 Dec 2020).

20 World Health Organization. Research for universal health coverage: World health report 2013. 2013. https://www.who.int/publications/i/item/9789240690837 (accessed 27 Jan

For peer review only

For peer review only - http://bmjopen.bmj.com/site/about/guidelines.xhtml

2021).

21 Franzen SRP, Chandler C, Lang T. Health research capacity development in low and middle income countries: Reality or rhetoric? A systematic meta-narrative review of the qualitative literature. BMJ Open. 2017;**7**:1–15. doi:10.1136/bmjopen-2016-012332

22 Magesa SM, Mwape B, Mboera LEG. Challenges and opportunities in building health research capacity in Tanzania: a case of the National Institute for Medical Research. *Tanzan J Health Res* 2012;**13**. doi:10.4314/thrb.v13i5.11

23 Yoshida S. Approaches, tools and methods used for setting priorities in health research in the 21st century. *J Glob Health* 2016;**6**:1–10. doi:10.7189/jogh.06.010507

24 World Health Organization. *A systematic approach for undertaking a research priority-setting exercise: guidance for WHO staff*. 2020. http://www.who.int/about/licensing. (accessed 22 Nov 2020).

25 Ditai J, Nakyazze M, Namutebi DA, *et al.* Maternal and newborn health priority setting partnership in rural Uganda in association with the James Lind Alliance: A study protocol. *Res Involv Engagem* 2020;**6**:57. doi:10.1186/s40900-020-00231-4

26 The James Lind Alliance. The James Lind Alliance Guidebook. 2020. www.jla.nihr.ac.uk (accessed 27 Nov 2020).

27 Bates I, Boyd A, Smith H, *et al.* A practical and systematic approach to organisational capacity strengthening for research in the health sector in Africa. *Heal Res Policy Syst* 2014;**12**:1–10. doi:10.1186/1478-4505-12-11

28 Munn Z, Peters MDJ, Stern C, *et al.* Systematic review or scoping review? Guidance for authors when choosing between a systematic or scoping review approach. *BMC Med Res Methodol* 2018;**18**:143. doi:10.1186/s12874-018-0611-x

For peer review only

For peer review only - http://bmjopen.bmj.com/site/about/guidelines.xhtml

29 Arksey H, O'Malley L. Scoping studies: Towards a methodological framework. *Int J Soc Res Methodol Theory Pract* 2005;**8**:19–32. doi:10.1080/1364557032000119616

30 Dow DE, Turner EL, Shayo AM, *et al.* Evaluating mental health difficulties and associated outcomes among HIV-positive adolescents in Tanzania. *AIDS Care - Psychol Socio-Medical Asp AIDS/HIV* 2016;**28**:825–33. doi:10.1080/09540121.2016.1139043

31 Rost L, Samuels F, Leon-Himmelstine C, *et al.* Digital approaches to adolescent mental health - a review of the literature. 2020. https://www.odi.org/sites/odi.org.uk/files/resource-documents/odi_digitalapproachesmentalhealth_final_1.pdf (accessed 15 Mar 2021).

32 Dow DE, Mmbaga BT, Turner EL, *et al.* Building resilience: a mental health intervention for Tanzanian youth living with HIV. *AIDS Care* 2018;**30**:12–20. doi:10.1080/09540121.2018.1527008

33 Kaaya S, Eustache E, Lapidos-Salaiz I, *et al.* Grand Challenges: Improving HIV Treatment Outcomes by Integrating Interventions for Co-Morbid Mental Illness. *PLoS Med* 2013;**10**:e1001447. doi:10.1371/journal.pmed.1001447

34 Auerswald CL, Piatt AA, Mirzazadeh A. Research with Disadvantaged, Vulnerable and/or Marginalized Adolescents. *Innocenti Res Briefs* 2017;**no. 06**.https://www.unicef-irc.org/publications/878-research-with-disadvantaged-vulnerable-and-or-marginalized-adolescents.html (accessed 15 Mar 2021).

35 Kuringe E, Materu J, Nyato D, *et al.* Prevalence and correlates of depression and anxiety symptoms among out-of-school adolescent girls and young women in Tanzania: A cross-sectional study. *PLoS One* 2019;**14**:e0221053. doi:10.1371/journal.pone.0221053

36 Liverpool School of Tropical Medicine. Data Protection Policy. 2018. https://www.lstmed.ac.uk/sites/default/files/centre/Data Protection Policy.pdf (accessed 23 Feb 2021).

For peer review only

For peer review only - http://bmjopen.bmj.com/site/about/guidelines.xhtml

37 National Institute for Medical Research. Information and Communication Technology Guidelines. 2016. https://www.nimr.or.tz/wp-content/uploads/2018/11/NIMR_ICT_GUIDELINES.pdf (accessed 23 Feb 2021).

38 ESSENCE on Health Research. Seven principles for strengthening research capacity in low- and middle-income countries: simple ideas in a complex world. World Health Organization 2014. https://www.who.int/tdr/publications/seven-principles/en/ (accessed 15 Mar 2021).

Figure legends:

Figure 1. The RPS process. Reproduced with permission from World Health Organization,[24] licence: CC BY-NC-SA 3.0 IGO.

Figure 2. Project flow diagram.

Figure 3. Survey and workshop recruitment flow chart.

For peer review only

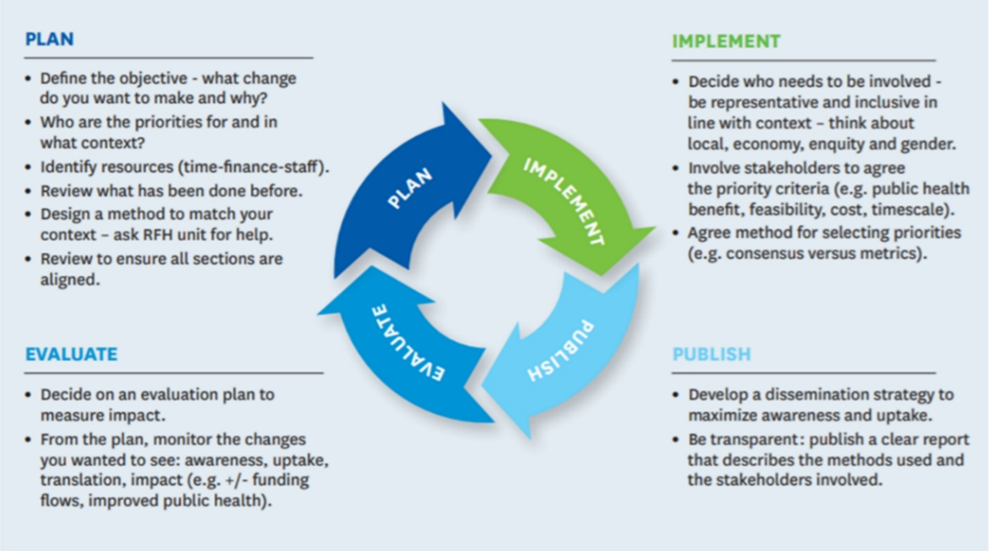

Figure 1. The RPS process. Reproduced with permission from World Health Organization,[24] licence: CC BY-NC-SA 3.0 IGO

350x193mm (87 x 87 DPI)

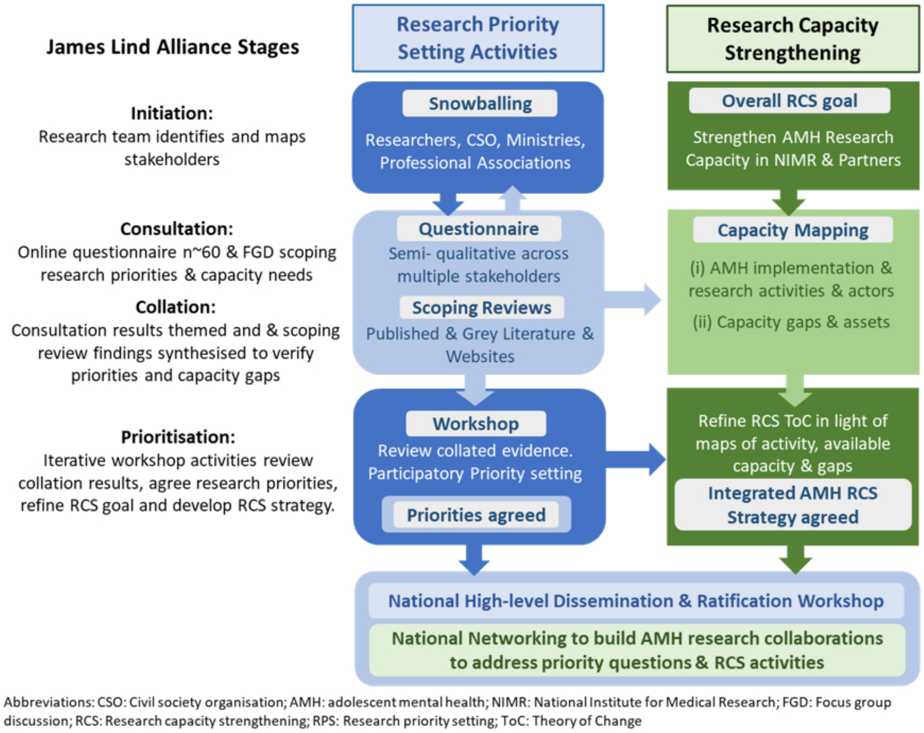

Figure 2. Project flow diagram

405x305mm (59 x 59 DPI)

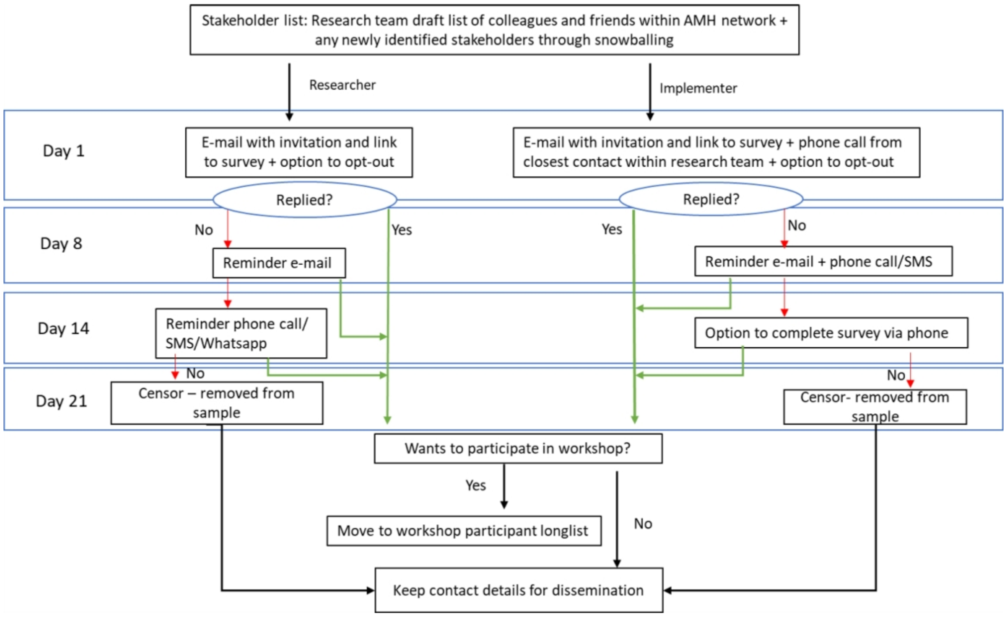

Figure 3. Survey and workshop recruitment flow chart.

658x401mm (59 x 59 DPI)

SPIRIT 2013 Checklist: Recommended items to address in a clinical trial protocol and related documents*

Section/item	Item No	Description	Addressed on page number
**Administrative information**			
Title	1	Descriptive title identifying the study design, population, interventions, and, if applicable, trial acronym	________1______
Trial registration	2a	Trial identifier and registry name. If not yet registered, name of intended registry	_______N/A___
	2b	All items from the World Health Organization Trial Registration Data Set	_____N/A__
Protocol version	3	Date and version identifier	_______N/A_____
Funding	4	Sources and types of financial, material, and other support	________22______
Roles and responsibilities	5a	Names, affiliations, and roles of protocol contributors	________1 &2_
	5b	Name and contact information for the trial sponsor	_____N/A_
	5c	Role of study sponsor and funders, if any, in study design; collection, management, analysis, and interpretation of data; writing of the report; and the decision to submit the report for publication, including whether they will have ultimate authority over any of these activities	_____22______
	5d	Composition, roles, and responsibilities of the coordinating centre, steering committee, endpoint adjudication committee, data management team, and other individuals or groups overseeing the trial, if applicable (see Item 21a for data monitoring committee)	____N/A_____

For peer review only

Introduction

Background and rationale	6a	Description of research question and justification for undertaking the trial, including summary of relevant studies (published and unpublished) examining benefits and harms for each intervention	_______6/7____
	6b	Explanation for choice of comparators	_____N/A_______
Objectives	7	Specific objectives or hypotheses	______7/8_____
Trial design	8	Description of trial design including type of trial (eg, parallel group, crossover, factorial, single group), allocation ratio, and framework (eg, superiority, equivalence, noninferiority, exploratory)	N/A___________

Methods: Participants, interventions, and outcomes

Study setting	9	Description of study settings (eg, community clinic, academic hospital) and list of countries where data will be collected. Reference to where list of study sites can be obtained	______10_________
Eligibility criteria	10	Inclusion and exclusion criteria for participants. If applicable, eligibility criteria for study centres and individuals who will perform the interventions (eg, surgeons, psychotherapists)	______10_________
Interventions	11a	Interventions for each group with sufficient detail to allow replication, including how and when they will be administered	______N/a__
	11b	Criteria for discontinuing or modifying allocated interventions for a given trial participant (eg, drug dose change in response to harms, participant request, or improving/worsening disease)	_______N/A_____
	11c	Strategies to improve adherence to intervention protocols, and any procedures for monitoring adherence (eg, drug tablet return, laboratory tests)	_____N/A________
	11d	Relevant concomitant care and interventions that are permitted or prohibited during the trial	_____N/A_______
Outcomes	12	Primary, secondary, and other outcomes, including the specific measurement variable (eg, systolic blood pressure), analysis metric (eg, change from baseline, final value, time to event), method of aggregation (eg, median, proportion), and time point for each outcome. Explanation of the clinical relevance of chosen efficacy and harm outcomes is strongly recommended	_____N/A________
Participant timeline	13	Time schedule of enrolment, interventions (including any run-ins and washouts), assessments, and visits for participants. A schematic diagram is highly recommended (see Figure)	____11/12_____

For peer review only - http://bmjopen.bmj.com/site/about/guidelines.xhtml

Sample size	14	Estimated number of participants needed to achieve study objectives and how it was determined, including clinical and statistical assumptions supporting any sample size calculations	___11/12_____
Recruitment	15	Strategies for achieving adequate participant enrolment to reach target sample size	____11_________

Methods: Assignment of interventions (for controlled trials)

Allocation:

Sequence generation	16a	Method of generating the allocation sequence (eg, computer-generated random numbers), and list of any factors for stratification. To reduce predictability of a random sequence, details of any planned restriction (eg, blocking) should be provided in a separate document that is unavailable to those who enrol participants or assign interventions	___N/A________
Allocation concealment mechanism	16b	Mechanism of implementing the allocation sequence (eg, central telephone; sequentially numbered, opaque, sealed envelopes), describing any steps to conceal the sequence until interventions are assigned	___N/A_______
Implementation	16c	Who will generate the allocation sequence, who will enrol participants, and who will assign participants to interventions	___N/A______
Blinding (masking)	17a	Who will be blinded after assignment to interventions (eg, trial participants, care providers, outcome assessors, data analysts), and how	___N/A______
	17b	If blinded, circumstances under which unblinding is permissible, and procedure for revealing a participant's allocated intervention during the trial	__N/A________

Methods: Data collection, management, and analysis

Data collection methods	18a	Plans for assessment and collection of outcome, baseline, and other trial data, including any related processes to promote data quality (eg, duplicate measurements, training of assessors) and a description of study instruments (eg, questionnaires, laboratory tests) along with their reliability and validity, if known. Reference to where data collection forms can be found, if not in the protocol	__11-14__
	18b	Plans to promote participant retention and complete follow-up, including list of any outcome data to be collected for participants who discontinue or deviate from intervention protocols	__N/A_____

Data management	19	Plans for data entry, coding, security, and storage, including any related processes to promote data quality (eg, double data entry; range checks for data values). Reference to where details of data management procedures can be found, if not in the protocol	___15______
Statistical methods	20a	Statistical methods for analysing primary and secondary outcomes. Reference to where other details of the statistical analysis plan can be found, if not in the protocol	____15______
	20b	Methods for any additional analyses (eg, subgroup and adjusted analyses)	_N/A_____
	20c	Definition of analysis population relating to protocol non-adherence (eg, as randomised analysis), and any statistical methods to handle missing data (eg, multiple imputation)	____N/A___
**Methods: Monitoring**			
Data monitoring	21a	Composition of data monitoring committee (DMC); summary of its role and reporting structure; statement of whether it is independent from the sponsor and competing interests; and reference to where further details about its charter can be found, if not in the protocol. Alternatively, an explanation of why a DMC is not needed	__N/A____
	21b	Description of any interim analyses and stopping guidelines, including who will have access to these interim results and make the final decision to terminate the trial	__N/A____
Harms	22	Plans for collecting, assessing, reporting, and managing solicited and spontaneously reported adverse events and other unintended effects of trial interventions or trial conduct	_N/A______
Auditing	23	Frequency and procedures for auditing trial conduct, if any, and whether the process will be independent from investigators and the sponsor	___N/A____
**Ethics and dissemination**			
Research ethics approval	24	Plans for seeking research ethics committee/institutional review board (REC/IRB) approval	______14_______
Protocol amendments	25	Plans for communicating important protocol modifications (eg, changes to eligibility criteria, outcomes, analyses) to relevant parties (eg, investigators, REC/IRBs, trial participants, trial registries, journals, regulators)	____N/A_____

Consent or assent	26a	Who will obtain informed consent or assent from potential trial participants or authorised surrogates, and how (see Item 32)	_______14__
	26b	Additional consent provisions for collection and use of participant data and biological specimens in ancillary studies, if applicable	_____N/A___
Confidentiality	27	How personal information about potential and enrolled participants will be collected, shared, and maintained in order to protect confidentiality before, during, and after the trial	_____14______
Declaration of interests	28	Financial and other competing interests for principal investigators for the overall trial and each study site	_____21_____
Access to data	29	Statement of who will have access to the final trial dataset, and disclosure of contractual agreements that limit such access for investigators	_____N/A_______
Ancillary and post-trial care	30	Provisions, if any, for ancillary and post-trial care, and for compensation to those who suffer harm from trial participation	________N/A___
Dissemination policy	31a	Plans for investigators and sponsor to communicate trial results to participants, healthcare professionals, the public, and other relevant groups (eg, via publication, reporting in results databases, or other data sharing arrangements), including any publication restrictions	_____15____
	31b	Authorship eligibility guidelines and any intended use of professional writers	__N/A_____
	31c	Plans, if any, for granting public access to the full protocol, participant-level dataset, and statistical code	_N/A_______
**Appendices**			
Informed consent materials	32	Model consent form and other related documentation given to participants and authorised surrogates	_____Appendix
Biological specimens	33	Plans for collection, laboratory evaluation, and storage of biological specimens for genetic or molecular analysis in the current trial and for future use in ancillary studies, if applicable	__N/A_____

*It is strongly recommended that this checklist be read in conjunction with the SPIRIT 2013 Explanation & Elaboration for important clarification on the items. Amendments to the protocol should be tracked and dated. The SPIRIT checklist is copyrighted by the SPIRIT Group under the Creative Commons "Attribution-NonCommercial-NoDerivs 3.0 Unported" license.

BMJ Open

Adolescent mental health research in Tanzania - a study protocol for a priority setting exercise and the development of an inter-institutional capacity strengthening programme.

Journal:	*BMJ Open*
Manuscript ID	bmjopen-2021-054163.R1
Article Type:	Protocol
Date Submitted by the Author:	04-Nov-2021
Complete List of Authors:	Obasi, Angela; Liverpool School of Tropical Medicine, International public health<br>Seekles, Maaike; Liverpool School of Tropical Medicine, Department of International Public Health<br>Boshe, Judith; Kilimanjaro Christian Medical Centre, Psychiatry and Mental Health; Kilimanjaro Christian Medical College, Psychiatry and Mental Health<br>Dow, Dorothy; Duke University Medical Center; Kilimanjaro Christian Medical Centre<br>Mmbaga, Blandina; Kilimanjaro Christian Medical Centre<br>Ngakongwa, Fileuka; Muhimbuli National Hospital, Department of Psychiatry and Mental Health<br>Okello, Elialilia; National Institute for Medical Research Mwanza Research Centre, Mwanza Intervention Trials Unit<br>Renju, Jenny; LSHTM; Kilimanjaro Christian Medical University College, Epidemiology and Biostatistics<br>Shayo, Elizabeth; National Institute for Medical Research<br>Simbee, Gema; Mirembe National Psychiatric Hospital<br>Todd, Jim; LSHTM; National Institute for Medical Research Mwanza Research Centre<br>Oriyo, Ndekya; National Institute for Medical Research
**Primary Subject Heading**:	Mental health
Secondary Subject Heading:	Global health, Public health
Keywords:	MENTAL HEALTH, Child & adolescent psychiatry < PSYCHIATRY, EDUCATION & TRAINING (see Medical Education & Training), Health policy < HEALTH SERVICES ADMINISTRATION & MANAGEMENT, International health services < HEALTH SERVICES ADMINISTRATION & MANAGEMENT

SCHOLARONE™
Manuscripts

For peer review only - http://bmjopen.bmj.com/site/about/guidelines.xhtml

Title:

Adolescent mental health research in Tanzania - a study protocol for a priority setting exercise and the development of an inter-institutional capacity strengthening programme.

Corresponding author:

Dr Maaike Seekles
17E Pembroke Place
Liverpool L3 5PH
Maaike.Seekles@lstmed.ac.uk

Authors

Dr Angela Obasi*
Department of International Public Health
Liverpool School of Tropical Medicine
Liverpool, United Kingdom

Dr Maaike Seekles*
Department of International Public Health
Liverpool School of Tropical Medicine
Liverpool, United Kingdom

Dr Judith Boshe
Kilimanjaro Christian Medical University College
Moshi, Tanzania

Dr Dorothy Dow
Department of Pediatrics, Infectious Diseases,
Duke University
Durham, North Carolina, USA

Professor Blandina Mmbaga
Kilimanjaro Clinical Research Institute
Moshi, Tanzania

Dr Fileuka Ngakongwa
Department of Psychiatry and Mental Health
Muhimbili National Hospital
Dar es Salaam, Tanzania

Dr Elialilia Okello
Mwanza Intervention Trials Unit
NIMR Mwanza Research centre, Mwanza, Tanzania

For peer review only

Dr Jenny Renju
Department of Epidemiology and Biostatistics,
Kilimanjaro Christian Medical University College
Moshi, Tanzania

Dr Elizabeth H. Shayo
Department of Policy Analysis and Advocacy
National Institute for Medical Research
Dar es Salaam, Tanzania

Dr Gema Simbee
Mirembe National Psychiatric Hospital
Dodoma, Tanzania

Professor Jim Todd
Department of Population Health
London School of Health and Tropical Medicine
London, United Kingdom

Dr Ndekya Oriyo[†]
National Institute for Medical Research
Dar es Salaam, Tanzania

***** Joint first authors

[†] Senior Author

Word count: 3630 words (incl declarations, excl abstract, figures, tables, references and appendix)

For peer review only

For peer review only - http://bmjopen.bmj.com/site/about/guidelines.xhtml

Abstract

Introduction

Poor adolescent mental health is a barrier to achieving several sustainable development goals in Tanzania, where adolescent mental health infrastructure is weak. This is compounded by a lack of community and policy maker awareness or understanding of its burden, causes and solutions. Research addressing these knowledge gaps is urgently needed. However, capacity for adolescent mental health research in Tanzania remains limited. The existence of a National Institute for Medical Research (NIMR), with a nationwide mandate for research conduct and oversight, presents an opportunity to catalyse activity in this neglected area. Rigorous research priority setting, which includes key stakeholders, can promote efficient use of limited resources and improve both quality and uptake of research by ensuring that it meets the needs of target populations and policy makers. We present a protocol for such a research priority setting study and how it informs the design of an inter-institutional adolescent mental health research capacity strengthening strategy in Tanzania.

Methods and analysis

From May 2021, this six-month mixed-methods study will adapt and merge the James Lind Alliance approach and validated capacity strengthening methodologies to identify priorities for research and research capacity strengthening in adolescent mental health in Tanzania. Specifically, it will use online questionnaires, face-to-face interviews, focus groups, scoping reviews and a consensus meeting to consult expert and adolescent stakeholders. Key evidence-informed priorities will be collaboratively ranked and documented and an integrated strategy to address capacity gaps will be designed to align with the nationwide infrastructure and overall strategy of NIMR.

Ethics and Dissemination

National and Institutional review board approvals were sought and granted from the National Health Research Ethics Committee of the NIMR Medical Research Coordinating Committee (Tanzania) and

For peer review only - http://bmjopen.bmj.com/site/about/guidelines.xhtml

the Liverpool School of Tropical Medicine (United Kingdom). Results will be disseminated through a national workshop involving all stakeholders, through ongoing collaborations and published commentaries, reviews, policy briefs, webinars and social media.

Key words: Mental health, adolescent health, capacity strengthening, research priority setting, Tanzania, James Lind Alliance

For peer review only

For peer review only - http://bmjopen.bmj.com/site/about/guidelines.xhtml

Strengths and limitations of this study

1. Systematic inclusion of all stakeholders, including policy makers
2. Nationwide approach and dissemination strategy using NIMR infrastructure
3. Transparent and integrated approach to research priority setting and capacity strengthening
4. Inclusion of adolescent stakeholders aged 18+ limits applicability to younger adolescents at this stage but will be the focus of future activity

For peer review only

For peer review only - http://bmjopen.bmj.com/site/about/guidelines.xhtml

INTRODUCTION

Mental health disorders account for a high burden of disease among young people worldwide. They are responsible for three of the top six causes of disability adjusted life years lost among 10-24 year olds [1] and affect up to 20% of all adolescents.[2] Suicide is the third leading cause of death in this age group. Adolescents who experience depressive disorders and other mental health problems are more likely to engage in risky behaviours, such as unprotected sexual activity and substance misuse. They are also more likely to have low educational attainment, to be unemployed and to live in poverty.[3] Much of adult mental ill-health begins in adolescence, with an estimated 75% of all lifetime mental disorders occurring by the age of 25.[4] The burden of mental ill-health among adolescents and its potential impact on adult outcomes makes adolescence a critical time for mental health promotion, early identification of mental health disorders, and rapid access to effective adolescent mental health (AMH) care.[2, 5, 6] This is especially important in Africa, which will be home to around 35 per cent of the world's adolescents by 2050.[7]

In Tanzania, where around half of the population is under 20 years of age, there are several important barriers to effective prevention and care for AMH disorders.[8] First, research on the prevalence, causes and solutions for several AMH issues is lacking.[9,10] The few available studies, mainly on depression and alcohol use disorders, indicate that AMH issues are common in Tanzania.[8,11] Second, AMH disorders are often not recognised as illnesses and stigmatisation of mental ill-health presents a further barrier to care.[12,13] Third, AMH is a low policy priority.[14] In 2017, there were 0.06 psychiatrists and 0.01 psychologists per 100,000 population; this is well below average for the Africa Region.[15] Currently, Tanzania has only one psychiatrist specialised in child and AMH disorders (Dr Gema Simbee, 2021). Further, primary and community-based healthcare providers, such as community health workers, general nurses, and community health officers, often have received very limited training, if any, in the delivery of mental healthcare to adolescents.[16, 17]

For peer review only - http://bmjopen.bmj.com/site/about/guidelines.xhtml

Health research is an essential tool for addressing health and development challenges and for informing health policy. Careful prioritisation of research to maximise its likely impact and the efficient use of resources is especially important where resources are scarce.[18] Of equal importance is the capacity to conduct high quality research to address identified priorities. Limited research capacity in low and middle income countries (LMIC) is a recognised barrier to improved health and health systems.[19–21] Research capacity in Tanzania faces both significant challenges and opportunities.[22] In particular, the existence of a national, publicly funded Institute for Medical Research provides the opportunity for systematic, nationwide scale up of both research and capacity strengthening. With eight research centres across Tanzania, The National Institute for Medical Research (NIMR) is the largest research Institute in Tanzania. It is mandated by government to conduct, oversee, and drive health research nationally, develop national health research priorities and oversee research implementation.

Against this background, the present study will first conduct rigorous research priority setting (RPS) to identify current AMH research gaps and priorities in Tanzania. In line with best practice, perspectives of policy makers, implementers and adolescent advocates will be thoroughly canvassed to ensure priorities meet the needs of all stakeholders. This will include mapping of AMH service implementation within the Government and civil society sector, including professional associations and internationally funded programmes. Second, we will concurrently map and identify gaps in existing AMH research capacity. This will inform the development of a strategy to ensure sufficient research capacity to address identified research gaps and priorities, including optimal use of the opportunity provided by NIMR and other research institutions and Civil Society Organisations (CSOs) within the country.

Study aims and objectives

The study aims are two-fold: first to identify gaps and priorities in AMH research in Tanzania which will inform the co-development of an evidence-based, stakeholder-driven research agenda; and

For peer review only - http://bmjopen.bmj.com/site/about/guidelines.xhtml

second to identify gaps in capacity needed to deliver this research agenda which will inform the development of a national research capacity strengthening (RCS) programme to address these gaps.

The specific research objectives are as follows:

1. Identify and bring together leading researchers, implementers and agencies, policy and decision-makers, and other AMH stakeholders in Tanzania.
2. Summarise and catalogue available evidence on the AMH burden and on the implementation and evaluation of AMH interventions in Tanzania.
3. Identify research priorities and develop a future research agenda for AMH.
4. Map existing AMH research capacity and identify gaps in capacity needed to deliver the research agenda.
5. Design an RCS programme to address AMH research priorities.

METHODS AND ANALYSIS

Rationale for selection of methodologies

There is considerable variation in approaches, tools and methods used in health RPS.[23] Recent WHO RPS guidance[24] identifies four phases from planning to evaluation (Figure 1) and discusses a range of tools and approaches which can be used in each phase. The guidance recommends use of comprehensive approaches to improve the quality and transparency of the exercise.[24] One widely-recommended approach, which has been used previously in Africa,[25] is a methodology designed by the James Lind Alliance (JLA).[26]

Insert here: Figure 1. The RPS process. Reproduced with permission from World Health Organization,[24] licence: CC BY-NC-SA 3.0 IGO.

Well-described and freely available, it consists of four steps which are aligned to the planning and implementation phases of the WHO cycle as follows (See Figure 2):

For peer review only - http://bmjopen.bmj.com/site/about/guidelines.xhtml

1. Initiation: Establish a group of stakeholders.
2. Gathering uncertainties (consultation): Via a questionnaire, stakeholders are asked to present their list of possible research priorities.
3. Verifying uncertainties (collation): The research team theme the possible priorities. A literature search is done to check whether there is any existing research evidence on the possible priorities.
4. Prioritisation: During a priority setting workshop, involving discussions in break-out groups and the stakeholder group as a whole, a top 10 of research priorities will be agreed upon.

The JLA methodology ensures a transparent and rigorous approach to research priority setting. However, it focuses specifically on identifying priorities for research on the effects of treatment interventions. Since our priority setting exercise has a broader scope, not all aspects of the JLA process are applicable, or possible. The current research will instead adapt and build on the JLA methodology and combine it with activities related to RCS as the second component of this study.

Our RCS will use methods recommended by Bates et al. which have been validated in a number of research institutions in Africa [27] and consist of the following steps,[27] the first four of which fall within the scope of this study:

1. Define the goal of the capacity strengthening programme.
2. Describe the required capacity needed to achieve the goal.
3. Determine the existing capacity and identify any gaps compared to the required capacity.
4. Devise and implement an action plan to fill the gaps, setting out activities, cost implications and indicators of progress.
5. Learn through doing; adapt the plan and indicators regularly.

This protocol details the application of the RPS and the modification of the RCS methods and processes that will be implemented to achieve the study objectives.

For peer review only

For peer review only - http://bmjopen.bmj.com/site/about/guidelines.xhtml

Detailed Study Design

This mixed methods study will be conducted over six months, from May to December 2021, in five overlapping phases. It will include online questionnaires, scoping reviews of the literature, individual interviews, focus group discussions and workshops. RPS and RCS activities will run concurrently where appropriate (See figure 2). This protocol has been prepared using the Standard Protocol Items: Recommendations for Interventional Trials Guidelines (SPIRIT, see supplementary file 1), where applicable.

Participants

All stakeholders in the field of AMH in Tanzania will be eligible to participate if they are 18 years of age or older. This includes active researchers, implementers, agencies, youth advocacy groups, and any relevant policy makers. For logistical reasons including prevailing COVID-19 restrictions and the lack of resources needed to create safe spaces for younger adolescents currently receiving treatment, adolescents aged below 18 years could not be included. However, this study will inform the design of a future priority setting activity that will focus specifically on younger adolescent services users and their carers.

Patient and public involvement

This preparatory consultation study is constrained by limited resources, the challenges of the ongoing COVID-19 pandemic and the complexities related to access to vulnerable AMH service users. The study largely applies the methods as described in the JLA RPS methodology, but departs from the approach in terms of study population. A JLA RPS exercise typically involves patients, carers and clinicians.[26] Our study is limited to professional stakeholders and youth advocates aged 18 or over. As explained above, engaging younger patients is outside the scope of this study, but will be a main subject of investigation in future follow-on studies when Covid-19 constraints have been relaxed and

For peer review only

For peer review only - http://bmjopen.bmj.com/site/about/guidelines.xhtml

funds are available to ensure safety and support. This study will, however, gain the input from young people and service users in the community by including representatives of youth advocacy groups, whose expert views are of pivotal importance.

Insert here: Figure 2. Project flow diagram.

Processes

Phase 1: Identification and recruitment of stakeholders (Spring 2021)

The initiation phase aims to identify and bring together stakeholders in the field of AMH. As a starting point, the research team, which includes researchers from NIMR, Kilimanjaro Christian Medical University College, Research Institute and Medical Centre and Muhimbili University of Health and Allied Sciences, will list any active researchers, implementers and agencies, and youth advocacy groups that they are aware of in Tanzania. In addition, relevant policy makers working in the field of AMH will be identified. The identification of stakeholders will be an iterative process throughout this study. As a project deliverable, a heat map will be created of identified AMH researchers and implementers across the country

Phase 2: Initial consultation (Summer 2021)

The second phase aims to 'gather uncertainties'[26] and gain an initial insight into stakeholder views on priorities and capacity gaps in AMH research.

Insert here: Figure 3. Survey and workshop recruitment flow chart.

Questionnaire

The research team will distribute a link to an on-line questionnaire (using Survey Monkey) to all identified stakeholders. It is expected that around 60 participants will be reached. A sample size calculation was not appropriate, since all identified stakeholders will be invited to participate. The questionnaire will

For peer review only - http://bmjopen.bmj.com/site/about/guidelines.xhtml

include a consent statement. This will be followed by a mix of open-ended and closed questions aimed at capturing the range and coverage of current AMH activities, in addition to priorities for research. The questions will be adapted slightly by type of stakeholder, but will generally address current involvement in the field of AMH service provision or research; perceived gaps in AMH research skills/capacity; and gaps in provision of AMH care. In addition, participants will be asked to list three AMH research priorities (see Supplementary file 1 for the questionnaire sent to researchers).

To ensure that all active stakeholders are reached through a process of snowballing, respondents will be asked to nominate other relevant stakeholders. Respondents will also be invited to signal their willingness to participate in the remainder of the RPS and RCS process. The data collection will take place over a period of one month commencing May 2021. In case of non-response, to maximise participation, identified stakeholders will receive two reminders via e-mail. Because of the high likelihood that stakeholders not supported by research infrastructure will have poor internet connectivity, non-researchers will receive reminders via phone calls and/or a text message. The study will also be publicised through research and implementer networks (see figure 3).

Once the survey is closed, members of the research team will come together to review, interpret, and sort participant priorities. During discussions, responses will be turned into a list of indicative questions for research, or ‘evidence uncertainties’.[26] These will be entered into a data management spreadsheet. Questions will be framed according to the Population, Intervention, Control, Outcome (PICO) format where possible. Out-of-scope submissions will be removed.

Phase 3: Data processing and verifying uncertainties

The JLA and Bates et al. recommend the use of a literature review in an early stage of RPS and RCS exercises.[26, 27] For the RPS, this is to verify that each indicative question identified in the survey is a true uncertainty. For each indicative question, a search in the Cochrane Database of Systematic Reviews and LSTM’s library resources will be done to identify systematic and scoping reviews that might address the uncertainty. Any recommendations for further research identified in the reviews

For peer review only - http://bmjopen.bmj.com/site/about/guidelines.xhtml

will be noted and may be included as priorities in the spreadsheet. Only up-to-date reviews, published in or after 2017, that focus on adolescent research in sub-Saharan Africa will be explored. For each research question, a Question Verification Form [26] will be completed to show how the research team concluded that the question is broadly unanswered.

Phase 4: Scoping reviews (Spring/Summer 2021)

Initial searches have already identified a gap in relevant AMH literature reviews in sub-Saharan Africa. In preparation of the priority setting exercise, we will undertake two scoping reviews, on AMH promotion/prevention and treatment interventions. A scoping review does not aim to produce a critically appraised and synthesised answer to a precise question related to a certain treatment or practice. It rather aims to provide an overview or map of the available evidence.[28] It can report on the types of evidence and the way research has been conducted, which aligns with the interests of the research team. The scoping reviews will follow the 5-step framework of Arksey and O'Malley.[29] They will include scientific and grey literature about studies with adolescents, undertaken in sub-Saharan Africa from 2000 onwards. Searches will be done in English and Kiswahili.

Phase 5: Prioritisation and RCS programme development (Autumn 2021)

The final phase aims to synthesise the information collected through the above activities and bring stakeholders together to design a research agenda and develop an RCS programme.

Workshop

After analysis of the questionnaire and initial scoping reviews, a workshop will synthesise research priorities and refine the goal for the RCS programme. Participants will be selected from the larger stakeholder group based on their availability and whether they registered an interest to take part. It is estimated that around 25 stakeholders, including national and sub-national representatives of the Ministry of Health and local government, will be able to participate. This falls within the range

For peer review only - http://bmjopen.bmj.com/site/about/guidelines.xhtml

recommended by the JLA of 12-30 participants per workshop.[26] Should more than 30 stakeholders want to participate, the research team will purposively select participants to ensure an appropriate representation of all stakeholder groups. During the two-day workshop, group discussions will take place, focusing on current research and perceived gaps in research, research capacity and care for AMH.

Per JLA guidance, an adapted Nominal Group Technique will be used as the method for research priority setting. Participants will first form smaller discussion groups of maximum eight participants, during which each participant gives their view about which priorities are most and least important, followed by shared ranking or voting. The ranked orders for each item from each group are totalled, and the priority with the lowest, i.e., most favoured, total ranking is selected as the top priority. The ranking is then discussed and reviewed in the large group, to arrive at a final list of 10 research priorities.[26] The final priorities will inform the development of a research agenda on AMH, with key research questions outlined in collaboration with partners.

The remainder of the workshop will focus on RCS. During group discussions, a process of joint problem-solving with stakeholders will identify strengths, prioritise critical capacity gaps, and transform what evidence indicated was optimal into what is feasible and practical. The gaps, reasons for the gaps, discrepancies, and resolutions, and potentially sustainable solutions for filling capacity gaps are mapped onto a matrix.[27] The team, together with participants, will then use this matrix to underpin the design of a theory of change and an RCS programme. The RCS strategy will align activities for strengthened capacity, e.g., mentoring, PhD in specific areas, skills development to potential studies and study partnerships, with identified research priorities. Intra and inter-institutional partnerships and additional resources needed to deliver these activities will be agreed.

During workshop days, a small number of key informant interviews (n~5-10) and focus group discussions (e.g., with representatives of national professional organisations and youth advocates) will take place, to verify identified priorities in light of local knowledge about context, epidemiology and ongoing

For peer review only - http://bmjopen.bmj.com/site/about/guidelines.xhtml

activities. Focus groups and interviews will be conducted in English or Kiswahili. They will be recorded and transcribed verbatim.

Ethics and Dissemination

National and Institutional review board approvals were sought and granted from the National Health Research Ethics Committee of the NIMR Medical Research Coordinating Committee in Tanzania (Reference number: NIMR/HQ/R.8a/Vol. IX/3670) and the LSTM in the United Kingdom (Reference number: 21-002).

Participation in this study is voluntary. All stakeholders will receive information about the study, to allow them to make an informed decision about their participation. Survey participants are asked to confirm approval with a consent statement. At the workshop, participants will be asked to sign an informed consent form. They have the right to withdraw at any time by informing the Principal Investigator. However, participants are advised that once their data is anonymised, it cannot be removed. Due to the nature of the focus group discussions and workshops, confidentiality cannot be guaranteed, but participants will be reminded to not share any sensitive data that can bring harm upon themselves or others.

A COVID-19 Risk Assessment, detailing appropriate precautions to limit the spread of COVID-19, has been completed. This will be updated throughout the study duration, to ensure that research activities comply with latest regulations.

A national dissemination meeting will be organised in Tanzania once the strategy and priority setting is finalised and written up, during which the results of the RPS exercise and RCS assessment will be presented. The developed national AMH research agenda will be launched as a platform for collaborative, inter-institutional research programmes which will embed RCS activities. The findings will be further disseminated through papers, commentaries, reviews, and policy briefs.

For peer review only - http://bmjopen.bmj.com/site/about/guidelines.xhtml

Data management and analysis

This study will comply with LSTM and NIMR data protection policies.[30,31] All electronic data and personal information of participants will be stored on password protected computers and secure drives, only accessed by designated members of the research team as appropriate. Group discussions and interviews will be recorded on an encrypted device; recordings will be transcribed verbatim as soon as possible after data collection, after which recordings will be deleted. Data will be stored for a maximum of 10 years, to allow verification of data from external sources if necessary, or longer if used for further research.

Data from online questionnaires will be extracted from Survey Monkey and simple descriptive statistical analysis will be undertaken, using Excel software to provide summaries of the sample and responses. In addition, answers to open questions will be coded and analysed through team discussions using content analysis to arrive at an initial list of gaps in research and capacities.

Workshop data and data collected during group discussions and interviews will be thematically analysed, using NVivo software, to offer a more in-depth account of arguments for or against research priorities, and explanations and possible solutions for capacity constraints and research gaps.

DISCUSSION

To summarise, this study will use a mixed-methods approach to consult experts, policy-makers and advocates and identify priorities and capacity gaps for AMH research in Tanzania. It gains its strengths from a multidisciplinary partnership between, and the involvement from inception of, key stakeholders from a number of research institutions across Tanzania; the use of a systematic, transparent approach to RPS; an evidence-based approach to RCS which is aligned to priority research activities; and a strong commitment to dissemination.

As discussed earlier, this study departs from the JLA method in terms of study population. It also departs from the approach in terms of scope of investigation. The JLA approach usually focuses on

For peer review only - http://bmjopen.bmj.com/site/about/guidelines.xhtml

uncertainties around treatment for a specific illness.[26] The aim of this study is to identify priorities on a national level, across the spectrum of AMH research. This could include uncertainties around prevalence, prevention, and treatment of varying illnesses, in addition to wider systemic challenges. By engaging key policy makers and the leadership and structure of the NIMR, it is hoped this study will raise the profile of AMH research within national research policy. The inter-institutional RCS partnerships generated through this process are also aligned to other best practice strategies for equitable partnership such as the WHO ESSENCE guidance.[32] In particular, it is hoped that the opportunities provided for AMH researchers, representatives from civil society organisations, development partners, youth advocates and senior government to meet and network during this study, will significantly strengthen AMH research structure and legacy.

For peer review only

DECLARATIONS

Consent for publication

Not applicable.

Competing interests

The authors declare that they have no competing interests.

Author's contributions

AO and NO are co- principal investigators who jointly secured funding for, initiated and led conception of the study. They share overall responsibility for the research. All authors (AO, NO, MS, JB, DD, BM, FN, EO, JR, ES, GS, and JT) attended project meetings, and contributed to the design of the study and data collection materials. As joint first authors, AO and MS led the drafting of the paper. All other authors (NO, JB, DD, BM, FN, EO, JR, ES, GS, and JT) provided critical input for revisions and approved the final version. With the exception of AO, NO and MS, authors are listed alphabetically.

For peer review only - http://bmjopen.bmj.com/site/about/guidelines.xhtml

Funding

This study is funded by a networking grant from the Global Challenges Research Fund (UKRI, Ref: GCRFNGR6_1454). The funder has no involvement in the design; collection, management, analysis, and interpretation of data; writing of the report, and the decision to submit the report for publication, nor do they have ultimate authority over any of these activities.

REFERENCES

1 Abbafati C, Machado DB, Cislaghi B, *et al.* Global burden of 369 diseases and injuries in 204 countries and territories, 1990–2019: a systematic analysis for the Global Burden of Disease Study 2019. *Lancet* 2020;**396**:1204–22. doi:10.1016/S0140-6736(20)30925-9

2 World Health Organization. Adolescent Mental Health: Time for Action. 2019. https://www.who.int/pmnch/knowledge/publications/AMH.pdf?ua=1 (accessed 1 Dec 2020).

3 Nyundo A, Manu A, Regan M, *et al.* Factors associated with depressive symptoms and suicidal ideation and behaviours amongst sub-Saharan African adolescents aged 10-19 years: cross-sectional study. *Trop Med Int Heal* 2020;**25**:54–69. doi:10.1111/tmi.13336

4 Kessler RC, Amminger GP, Aguilar-Gaxiola S, *et al.* Age of onset of mental disorders: A review of recent literature. Curr. Opin. Psychiatry. 2007;**20**:359–64. doi:10.1097/YCO.0b013e32816ebc8c

5 World Health Organization. mhGAP Mental Health Gap Action Programme. World Health Organization 2013. https://www.who.int/mental_health/evidence/mhGAP/en/ (accessed 10 Dec 2020).

6 World Health Organization. Global Accelerated Action for the Health of Adolescents (AA-HA!) Guidance to Support Country Implementation. 2017.

For peer review only

For peer review only - http://bmjopen.bmj.com/site/about/guidelines.xhtml

https://apps.who.int/iris/bitstream/handle/10665/255415/9789241512343-eng.pdf?sequence=1 (accessed 10 Dec 2020).

7 UNICEF. Generation 2030 Africa 2.0 . 2017. https://data.unicef.org/resources/generation-2030-africa-2-0/ (accessed 26 Jan 2021).

8 Kutcher S, Perkins K, Gilberds H, *et al.* Creating Evidence-Based Youth Mental Health Policy in Sub-Saharan Africa: A Description of the Integrated Approach to Addressing the Issue of Youth Depression in Malawi and Tanzania. *Front Psychiatry* 2019;**10**:542. doi:10.3389/fpsyt.2019.00542

9 Cortina MA, Sodha A, Fazel M, *et al.* Prevalence of child mental health problems in Sub-Saharan Africa: A systematic review. Arch. Pediatr. Adolesc. Med. 2012;**166**:276–81. doi:10.1001/archpediatrics.2011.592

10 Kieling C, Baker-Henningham H, Belfer M, *et al.* Child and adolescent mental health worldwide: evidence for action. *Lancet* 2011;**378**:1515–25. doi:10.1016/S0140

11 Francis JM, Weiss HA, Mshana G, *et al.* The epidemiology of alcohol use and alcohol use disorders among young people in Northern Tanzania. *PLoS One* 2015;**10**. doi:10.1371/journal.pone.0140041

12 Iseselo MK, Kajula L, Yahya-Malima KI. The psychosocial problems of families caring for relatives with mental illnesses and their coping strategies: A qualitative urban based study in Dar es Salaam, Tanzania. *BMC Psychiatry* 2016;**16**:1–12. doi:10.1186/s12888-016-0857-y

13 Benedicto M, Mndeme E, Mwakagile DSM, *et al.* Community Knowledge, Attitudes and Perception towards Mental Illness in Dodoma Municipality, Tanzania. *ARC J Public Heal Community Med* 2016;**1**:10–8. doi:10.20431/2456-0596.0103002

14 Zhou W, Ouyang F, Nergui O-E, *et al.* Child and Adolescent Mental Health Policy in Low- and Middle-Income Countries: Challenges and Lessons for Policy Development and

For peer review only

For peer review only - http://bmjopen.bmj.com/site/about/guidelines.xhtml

Implementation. *Front Psychiatry* 2020;**11**:150. doi:10.3389/fpsyt.2020.00150

15 World Health Organization. Mental Health Atlas. 2018. https://apps.who.int/iris/bitstream/handle/10665/272735/9789241514019-eng.pdf?ua=1 (accessed 26 Jan 2021).

16 Kutcher S, Wei Y, Gilberds H, *et al.* Addressing Adolescent Depression in Tanzania: Positive Primary Care Workforce Outcomes Using a Training Cascade Model. *Depress Res Treat* 2017;**2017**:1–9. doi:10.1155/2017/9109086

17 World Health Organization. Mental Health Atlas 2017 - Republic of Tanzania. 2017. https://www.who.int/mental_health/evidence/atlas/profiles-2017/TZ.pdf?ua=1 (accessed 25 Nov 2020).

18 McGregor S, Henderson KJ, Kaldor JM. How are health research priorities set in low and middle income countries? A systematic review of published reports. *PLoS One* 2014;**9**:1–9. doi:10.1371/journal.pone.0108787

19 Council on Health Research for Development. Health Research: Essential Link to Equity in Development . 1990. http://www.cohred.org/publications/open-archive/1990-commission-report/ (accessed 11 Dec 2020).

20 World Health Organization. Research for universal health coverage: World health report 2013. 2013. https://www.who.int/publications/i/item/9789240690837 (accessed 27 Jan 2021).

21 Franzen SRP, Chandler C, Lang T. Health research capacity development in low and middle income countries: Reality or rhetoric? A systematic meta-narrative review of the qualitative literature. BMJ Open. 2017;**7**:1–15. doi:10.1136/bmjopen-2016-012332

22 Magesa SM, Mwape B, Mboera LEG. Challenges and opportunities in building health research capacity in Tanzania: a case of the National Institute for Medical Research. *Tanzan J Health*

For peer review only

For peer review only - http://bmjopen.bmj.com/site/about/guidelines.xhtml

Res 2012;**13**. doi:10.4314/thrb.v13i5.11

23 Yoshida S. Approaches, tools and methods used for setting priorities in health research in the 21st century. *J Glob Health* 2016;**6**:1–10. doi:10.7189/jogh.06.010507

24 World Health Organization. *A systematic approach for undertaking a research priority-setting exercise: guidance for WHO staff*. 2020. http://www.who.int/about/licensing. (accessed 22 Nov 2020).

25 Ditai J, Nakyazze M, Namutebi DA, *et al.* Maternal and newborn health priority setting partnership in rural Uganda in association with the James Lind Alliance: A study protocol. *Res Involv Engagem* 2020;**6**:57. doi:10.1186/s40900-020-00231-4

26 The James Lind Alliance. The James Lind Alliance Guidebook. 2020. www.jla.nihr.ac.uk (accessed 27 Nov 2020).

27 Bates I, Boyd A, Smith H, *et al.* A practical and systematic approach to organisational capacity strengthening for research in the health sector in Africa. *Heal Res Policy Syst* 2014;**12**:1–10. doi:10.1186/1478-4505-12-11

28 Munn Z, Peters MDJ, Stern C, *et al.* Systematic review or scoping review? Guidance for authors when choosing between a systematic or scoping review approach. *BMC Med Res Methodol* 2018;**18**:143. doi:10.1186/s12874-018-0611-x

29 Arksey H, O'Malley L. Scoping studies: Towards a methodological framework. *Int J Soc Res Methodol Theory Pract* 2005;**8**:19–32. doi:10.1080/1364557032000119616

30 Liverpool School of Tropical Medicine. Data Protection Policy. 2018. https://www.lstmed.ac.uk/sites/default/files/centre/Data Protection Policy.pdf (accessed 23 Feb 2021).

31 National Institute for Medical Research. Information and Communication Technology

For peer review only

For peer review only - http://bmjopen.bmj.com/site/about/guidelines.xhtml

Guidelines. 2016. https://www.nimr.or.tz/wp-content/uploads/2018/11/NIMR_ICT_GUIDELINES.pdf (accessed 23 Feb 2021).

32 ESSENCE on Health Research. Seven principles for strengthening research capacity in low- and middle-income countries: simple ideas in a complex world. World Health Organization 2014. https://www.who.int/tdr/publications/seven-principles/en/ (accessed 15 Mar 2021)

Figure legends:

Figure 1. The RPS process. Reproduced with permission from World Health Organization,[24] licence: CC BY-NC-SA 3.0 IGO.

Figure 2. Project flow diagram.

Figure 3. Survey and workshop recruitment flow chart.

Supplementary files:

Supplementary file 1. Research priority setting questionnaire – researcher version

For peer review only

For peer review only - http://bmjopen.bmj.com/site/about/guidelines.xhtml

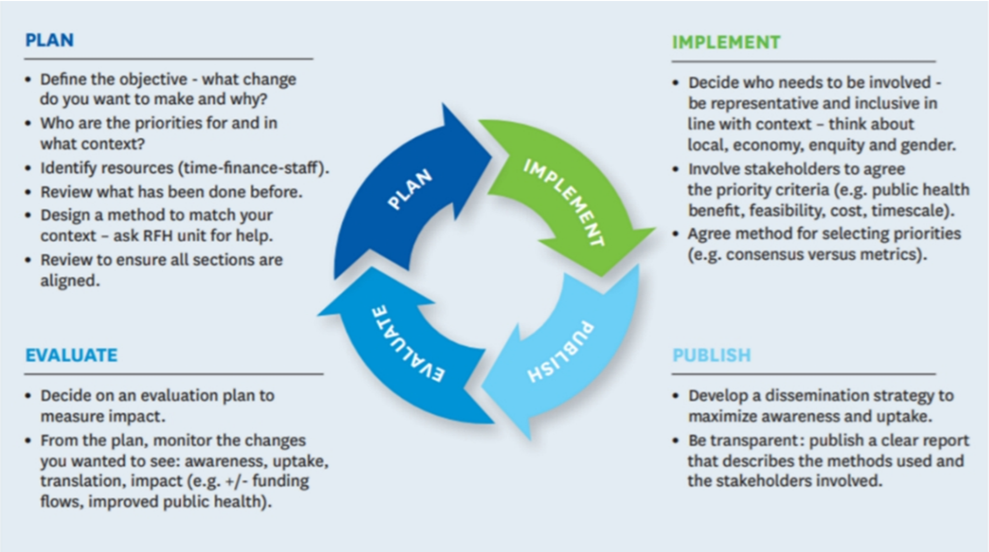

Figure 1. The RPS process. Reproduced with permission from World Health Organization,[24] licence: CC BY-NC-SA 3.0 IGO

350x193mm (87 x 87 DPI)

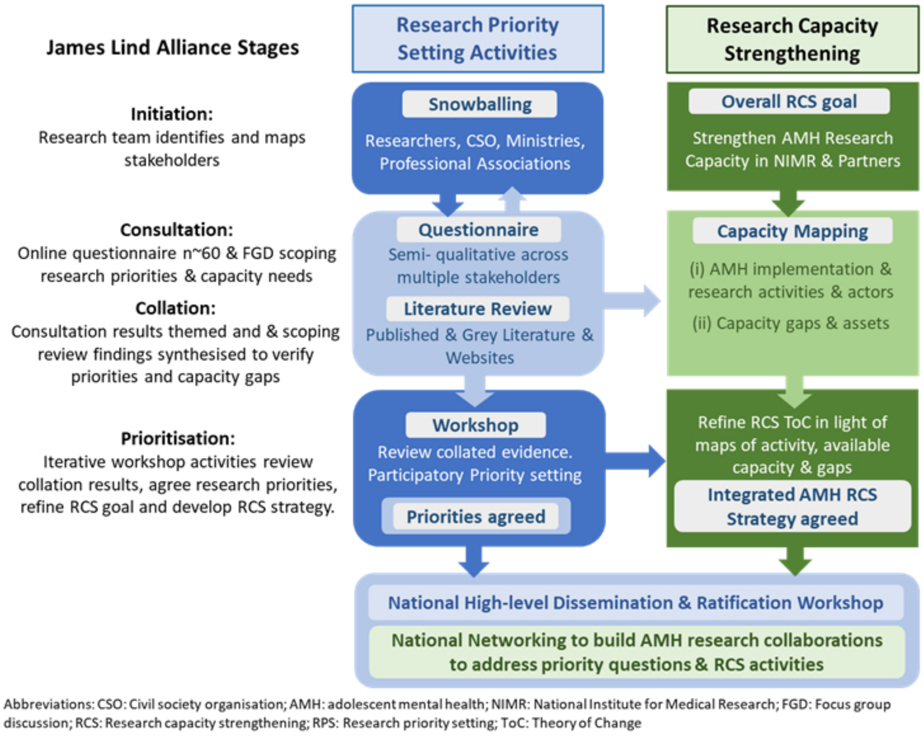

Figure 2. Project flow diagram.

350x264mm (57 x 57 DPI)

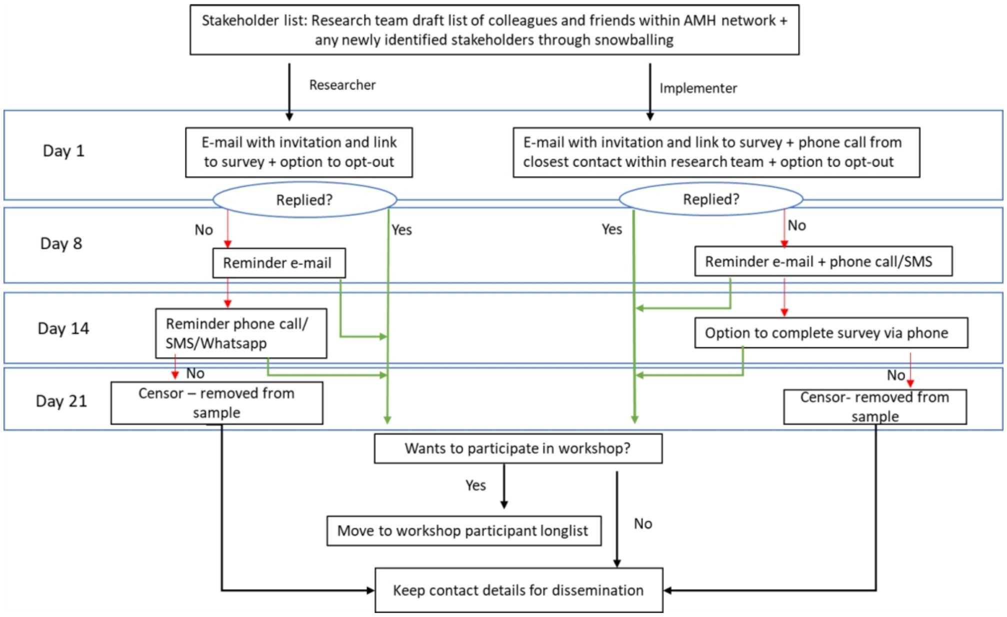

Figure 3. Survey and workshop recruitment flow chart.

658x401mm (59 x 59 DPI)

Scoping questionnaire to inform priority setting for adolescent mental health research in Tanzania:

Researcher Questionnaire v1.3

Study Information:

The National Institute of Medical Research (NIMR) in collaboration with researchers at the Liverpool School of Tropical Medicine, Kilimanjaro Christian Medical University College, and Muhimbili University of Health and Allied Sciences is conducting a research priority setting exercise for adolescent mental health in Tanzania. This involves consulting researchers, implementors and adolescent advocacy groups involved in adolescent mental health.

Why are we contacting you? We are contacting you because we understand that you work in adolescent health and could make an important contribution to this research priority setting exercise.

What do we need from you? A few minutes of your time to complete this short questionnaire about your work and views about adolescent mental health research in Tanzania.

Confidentiality: Your contribution will be treated with the strictest confidence. Your answers will be anonymised before analysis by the research team. Your name or contact information will not be linked to your responses. However at the end of the questionnaire you can leave contact details if you wish to participate in future activities. This contact information will be kept separate from your responses.

Voluntary participation: We would really value your contribution; it will improve the quality of our work. However, your participation is absolutely voluntary and you can withdraw at any time.

How long will the survey take? On average, 15 minutes.

Please feel free to contact us if you would like further information about the project.

Project co-Principal Investigators:

· Dr Ndekya Oriyo, Principal Research Scientist, The National Institute of Medical Research NIMR, Tanzania: ndekya.oriyo@nimr.or.tz

· Dr Angela Obasi, Dept International Public Health, The Liverpool School of Tropical Medicine, UK: angela.obasi@lstmed.ac.uk

---

CONSENT

I have read and understood the information about this survey and understand its purpose.

I understand that no identifiable information about me will be retained from this survey.

I understand that my participation is entirely voluntary and that I can stop or withdraw my participation at any time.

I agree to participate in the survey.

Yes ☐ No ☐

For peer review only - http://bmjopen.bmj.com/site/about/guidelines.xhtml

ONLINE QUESTIONS for Researcher Scoping Questionnaire

The questionnaire is in two parts. The first few questions are about the work that you are currently doing. The second set of questions ask about what you think are important gaps in adolescent mental health research and service provision.

PART 1: ABOUT THE WORK THAT YOU DO

1. **What is your gender?**
2. **Which age category do you belong to?**
3. **Which institution is your main employer?**

___

4. **Through which institution do you mainly conduct your research?**

___

The next question is about the main focus of your research activities in relation to adolescents

5. **What is your *primary or main* adolescent health research activity?** (Please choose one)**.**
   i. HIV prevention
   ii. HIV treatment and care
   iii. Sexual and reproductive health
   iv. Teenage pregnancy prevention
   v. Teenage pregnancy care
   vi. Adolescent mental health
   vii. Other, please explain

___

The next question is about your adolescent mental health research activities.

6. **MAIN STEM: Are you currently (or have you within the last 5 years been) involved in conducting research addressing adolescent mental health in Tanzania?**
   - Yes
   - No

   If YES (main stem)

7. **What is the primary area of adolescent mental health you are/were researching?**

___

For peer review only - http://bmjopen.bmj.com/site/about/guidelines.xhtml

a. What is/was the objective of this research

____

8. Which of the following best describes the areas that your <u>adolescent mental health</u> research overall is addressing? Activities are grouped by prevention or treatment. (Please click all that apply).

i. Prevention of alcohol use
ii. Prevention of non-alcoholic substance use
iii. Prevention of common mental health disorders e.g. depression, anxiety, PTSD
iv. Prevention of severe mental illness e.g schizophrenia or mania
v. Prevention of developmental disorders (ADHD, FASD, etc)

vi. Treatment for alcohol misuse
vii. Treatment for non-alcoholic substance use
viii. Treatment of common mental health disorders e.g. depression, anxiety, PTSD
ix. Treatment of severe mental illness e.g. schizophrenia or mania
x. Treatment of developmental disorders (ADHD, FASD, etc)

xi. Other, please explain

9. What is/was your role in this research

a. PI or Co-PI of one or more research projects in this area
b. Co- investigator of one or more research projects in this area
c. Research treatment doctor
d. Research assistant
e. Other

____

10. What setting is the primary focus of your studies ? (Please select ONE)

b. Adolescents attending mental health services
c. Adolescents in non-mental health clinical settings
d. Adolescents in primary school
e. Adolescents in secondary school
f. Adolescents attending college
g. Out of school/community-based
h. Adolescents in the workplace
i. Other? Please specify.

11. In terms of gender, what adolescent subgroup is the primary focus of your research?

a. Female adolescents/young people
b. Male adolescents/young people

For peer review only - http://bmjopen.bmj.com/site/about/guidelines.xhtml

c. Adolescents of any gender

12. What adolescent subgroup is the primary focus of your study? (Please select ONE).

a. HIV positive adolescents
b. Adolescents with disabilities
c. Adolescents who are refugees or seeking asylum
d. Adolescents in domestic service
e. Adolescents who are orphaned
f. Street connected youth
g. Any adolescent
h. Other? [please specify]

____

13. Are there additional adolescents, beyond the primary subgroup chosen above, that your research include? (Please select all that apply)

a. Not applicable
b. Adolescents attending mental health services
c. Adolescents in non mental health clinical settings
d. Adolescents in primary school
e. Adolescents in secondary school
f. Adolescents attending college
g. Out of school/community-based
h. Adolescents in the workplace
i. HIV positive adolescents
j. Adolescents who are orphaned
k. Adolescents with disabilities
l. Adolescents who are refugees or seeking asylum
m. Adolescents in domestic service
n. Street connected youth
o. Other? [please specify]

14. Where is your adolescent mental health research work located? (Click all that apply)

p. [insert all the regions]

____

15. Which of the following best describes your research activity? (click all that apply)

a. Understanding prevalence of mental ill-health among adolescents
b. Exploring risk factors for mental ill-health among adolescents
c. Development or evaluation of prevention interventions
d. Development or evaluation non-pharmacological treatment interventions
e. Development or evaluation pharmacological treatment interventions
f. Adolescent mental health programme evaluation or monitoring
g. Validating measures of adolescent mental ill health
h. Other [please specify]

____

16. Are there any key references, reports or summaries relating to your activities that you could share. If so please could you cite or paste in a weblink here.

For peer review only - http://bmjopen.bmj.com/site/about/guidelines.xhtml

13. In addition to your research work are you also involved in the provision of services or care that relate to AMH?

a. **YES (continue to 14)**
b. **No – go to the end section**

14. What is your *primary or main* adolescent health activity? (Please choose one)**.**

i. HIV prevention
ii. HIV treatment and care
iii. Sexual and reproductive health
iv. Teenage pregnancy prevention
v. Teenage pregnancy care
vi. Adolescent mental health
vii. Other, please explain

15. Which of the following best describes the type of adolescent mental health activities that you provide? (Click all that apply)

i. Counselling by trained mental health professionals
ii. Peer to peer support
iii. Biomedical treatment
iv. Behavioural treatment
v. Education/promotion/prevention
vi. Other, please explain

Main Stem if Q4 = NO

16. Have research questions relating to adolescent mental health arisen as part of your research activities

- Yes
- No

If yes, please specify

For peer review only - http://bmjopen.bmj.com/site/about/guidelines.xhtml

All stems converge here

PART 2

The next few questions are about what you think are important gaps in adolescent mental health research and service provision

17. Which 3 *areas/issues* in adolescent mental health do you think most urgently need further research?

____

____

____

a. Of these, which is most important for Tanzania as a whole?

____

b. Would you be interested in developing or building collaborations to address any of these research priorities?
   - Yes
   - No

c. If yes, which area?

____

18. Do you feel that there are gaps in skills/capacity to conduct adolescent mental health research
- **Yes**
- **No**

If yes, which gaps do you feel are the most important?

19. Do you feel that there are gaps in skills/capacity for provision of adolescent mental health care/services?
- **Yes**
- **No**

If yes, which gaps do you feel are the most important?

20. What, if any, networks/groups are you involved in that are conducting or related to Adolescent mental health research in Tanzania?

For peer review only - http://bmjopen.bmj.com/site/about/guidelines.xhtml

21. **Would you be interested in participating with us in further adolescent mental health research priority setting activities?**
    - **Yes**
    - **No**

If yes, please provide us with the best email and/or telephone contact for us to reach you

22. **Are there any other people working in Adolescent Mental Health that you feel we should approach to ask these questions?**

23. **Please feel free to use the box below to make any further comments, or share your reflections relating to adolescent mental health research in Tanzania.**

END OF QUESTIONNAIRE:

Thank you so much for your participation in this survey

We greatly appreciate your time

For any further queries please contact

ndekya,oriyo@nimr.or.tz or angela.obasi@lstmed.ac.uk

For peer review only - http://bmjopen.bmj.com/site/about/guidelines.xhtml

SPIRIT

STANDARD PROTOCOL ITEMS: RECOMMENDATIONS FOR INTERVENTIONAL TRIALS

SPIRIT 2013 Checklist: Recommended items to address in a clinical trial protocol and related documents*

Section/item	Item No	Description	Addressed on page number
**Administrative information**			
Title	1	Descriptive title identifying the study design, population, interventions, and, if applicable, trial acronym	_______1______
Trial registration	2a	Trial identifier and registry name. If not yet registered, name of intended registry	______N/A___
	2b	All items from the World Health Organization Trial Registration Data Set	_____N/A__
Protocol version	3	Date and version identifier	______N/A_____
Funding	4	Sources and types of financial, material, and other support	_______18______
Roles and responsibilities	5a	Names, affiliations, and roles of protocol contributors	_______1 &2_
	5b	Name and contact information for the trial sponsor	_____N/A_
	5c	Role of study sponsor and funders, if any, in study design; collection, management, analysis, and interpretation of data; writing of the report; and the decision to submit the report for publication, including whether they will have ultimate authority over any of these activities	_____18______
	5d	Composition, roles, and responsibilities of the coordinating centre, steering committee, endpoint adjudication committee, data management team, and other individuals or groups overseeing the trial, if applicable (see Item 21a for data monitoring committee)	____N/A_____

For peer review only

Introduction

Background and rationale	6a	Description of research question and justification for undertaking the trial, including summary of relevant studies (published and unpublished) examining benefits and harms for each intervention	______6/7____
	6b	Explanation for choice of comparators	____N/A______
Objectives	7	Specific objectives or hypotheses	_____7/8_____
Trial design	8	Description of trial design including type of trial (eg, parallel group, crossover, factorial, single group), allocation ratio, and framework (eg, superiority, equivalence, noninferiority, exploratory)	N/A__________

Methods: Participants, interventions, and outcomes

Study setting	9	Description of study settings (eg, community clinic, academic hospital) and list of countries where data will be collected. Reference to where list of study sites can be obtained	_____10________
Eligibility criteria	10	Inclusion and exclusion criteria for participants. If applicable, eligibility criteria for study centres and individuals who will perform the interventions (eg, surgeons, psychotherapists)	_____10________
Interventions	11a	Interventions for each group with sufficient detail to allow replication, including how and when they will be administered	_____N/a__
	11b	Criteria for discontinuing or modifying allocated interventions for a given trial participant (eg, drug dose change in response to harms, participant request, or improving/worsening disease)	______N/A_____
	11c	Strategies to improve adherence to intervention protocols, and any procedures for monitoring adherence (eg, drug tablet return, laboratory tests)	____N/A_______
	11d	Relevant concomitant care and interventions that are permitted or prohibited during the trial	____N/A______
Outcomes	12	Primary, secondary, and other outcomes, including the specific measurement variable (eg, systolic blood pressure), analysis metric (eg, change from baseline, final value, time to event), method of aggregation (eg, median, proportion), and time point for each outcome. Explanation of the clinical relevance of chosen efficacy and harm outcomes is strongly recommended	____N/A_______
Participant timeline	13	Time schedule of enrolment, interventions (including any run-ins and washouts), assessments, and visits for participants. A schematic diagram is highly recommended (see Figure)	___11/12_____

For peer review only - http://bmjopen.bmj.com/site/about/guidelines.xhtml

Sample size	14	Estimated number of participants needed to achieve study objectives and how it was determined, including clinical and statistical assumptions supporting any sample size calculations	___11/12_____
Recruitment	15	Strategies for achieving adequate participant enrolment to reach target sample size	____11_________

Methods: Assignment of interventions (for controlled trials)

Allocation:

Sequence generation	16a	Method of generating the allocation sequence (eg, computer-generated random numbers), and list of any factors for stratification. To reduce predictability of a random sequence, details of any planned restriction (eg, blocking) should be provided in a separate document that is unavailable to those who enrol participants or assign interventions	___N/A________
Allocation concealment mechanism	16b	Mechanism of implementing the allocation sequence (eg, central telephone; sequentially numbered, opaque, sealed envelopes), describing any steps to conceal the sequence until interventions are assigned	___N/A_______
Implementation	16c	Who will generate the allocation sequence, who will enrol participants, and who will assign participants to interventions	___N/A______
Blinding (masking)	17a	Who will be blinded after assignment to interventions (eg, trial participants, care providers, outcome assessors, data analysts), and how	___N/A______
	17b	If blinded, circumstances under which unblinding is permissible, and procedure for revealing a participant's allocated intervention during the trial	__N/A________

Methods: Data collection, management, and analysis

Data collection methods	18a	Plans for assessment and collection of outcome, baseline, and other trial data, including any related processes to promote data quality (eg, duplicate measurements, training of assessors) and a description of study instruments (eg, questionnaires, laboratory tests) along with their reliability and validity, if known. Reference to where data collection forms can be found, if not in the protocol	__11-14__
	18b	Plans to promote participant retention and complete follow-up, including list of any outcome data to be collected for participants who discontinue or deviate from intervention protocols	__N/A_____

Data management	19	Plans for data entry, coding, security, and storage, including any related processes to promote data quality (eg, double data entry; range checks for data values). Reference to where details of data management procedures can be found, if not in the protocol	___16______
Statistical methods	20a	Statistical methods for analysing primary and secondary outcomes. Reference to where other details of the statistical analysis plan can be found, if not in the protocol	____16______
	20b	Methods for any additional analyses (eg, subgroup and adjusted analyses)	_N/A_____
	20c	Definition of analysis population relating to protocol non-adherence (eg, as randomised analysis), and any statistical methods to handle missing data (eg, multiple imputation)	____N/A___

Methods: Monitoring

Data monitoring	21a	Composition of data monitoring committee (DMC); summary of its role and reporting structure; statement of whether it is independent from the sponsor and competing interests; and reference to where further details about its charter can be found, if not in the protocol. Alternatively, an explanation of why a DMC is not needed	__N/A____
	21b	Description of any interim analyses and stopping guidelines, including who will have access to these interim results and make the final decision to terminate the trial	__N/A____
Harms	22	Plans for collecting, assessing, reporting, and managing solicited and spontaneously reported adverse events and other unintended effects of trial interventions or trial conduct	_N/A______
Auditing	23	Frequency and procedures for auditing trial conduct, if any, and whether the process will be independent from investigators and the sponsor	___N/A____

Ethics and dissemination

Research ethics approval	24	Plans for seeking research ethics committee/institutional review board (REC/IRB) approval	______15_______
Protocol amendments	25	Plans for communicating important protocol modifications (eg, changes to eligibility criteria, outcomes, analyses) to relevant parties (eg, investigators, REC/IRBs, trial participants, trial registries, journals, regulators)	____N/A_____

Consent or assent	26a	Who will obtain informed consent or assent from potential trial participants or authorised surrogates, and how (see Item 32)	______15__
	26b	Additional consent provisions for collection and use of participant data and biological specimens in ancillary studies, if applicable	____N/A___
Confidentiality	27	How personal information about potential and enrolled participants will be collected, shared, and maintained in order to protect confidentiality before, during, and after the trial	_____16______
Declaration of interests	28	Financial and other competing interests for principal investigators for the overall trial and each study site	____17_____
Access to data	29	Statement of who will have access to the final trial dataset, and disclosure of contractual agreements that limit such access for investigators	____N/A_______
Ancillary and post-trial care	30	Provisions, if any, for ancillary and post-trial care, and for compensation to those who suffer harm from trial participation	_______N/A___
Dissemination policy	31a	Plans for investigators and sponsor to communicate trial results to participants, healthcare professionals, the public, and other relevant groups (eg, via publication, reporting in results databases, or other data sharing arrangements), including any publication restrictions	____15____
	31b	Authorship eligibility guidelines and any intended use of professional writers	__N/A_____
	31c	Plans, if any, for granting public access to the full protocol, participant-level dataset, and statistical code	_N/A_______
**Appendices**			
Informed consent materials	32	Model consent form and other related documentation given to participants and authorised surrogates	_____Appendix
Biological specimens	33	Plans for collection, laboratory evaluation, and storage of biological specimens for genetic or molecular analysis in the current trial and for future use in ancillary studies, if applicable	__N/A_____

*It is strongly recommended that this checklist be read in conjunction with the SPIRIT 2013 Explanation & Elaboration for important clarification on the items. Amendments to the protocol should be tracked and dated. The SPIRIT checklist is copyrighted by the SPIRIT Group under the Creative Commons "Attribution-NonCommercial-NoDerivs 3.0 Unported" license.
